# Changes in antibiotic prophylaxis prescribing and infective endocarditis admissions in England and Scotland following changes in national guidance: a quasi-experimental longitudinal study

**DOI:** 10.1016/j.lanepe.2026.101923

**Published:** 2026-11-07

**Authors:** Martin H. Thornhill, Bernard Prendergast, Mark Dayer, Peter Lockhart, Larry M. Baddour

**Affiliations:** a School of Clinical Dentistry, University of Sheffield, Sheffield, UK; b Cleveland Clinic, London, UK; c Cardiovascular Research Institute, Mater Private Network, Dublin, Ireland; d Faculty of Health, University of Plymouth, Plymouth, UK; e Department of Oral Medicine/Oral & Maxillofacial Surgery, Wake Forest University School of Medicine, Atrium Health – Carolinas Medical Center, Charlotte, NC, USA; f Division of Public Health, Infectious Diseases, and Occupational Health, Departments of Medicine and Cardiovascular Medicine, Mayo Clinic College of Medicine and Science, Rochester, MN, USA

**Keywords:** Infective endocarditis, Antibiotic prophylaxis, Guidelines, Invasive dental procedures, Prevention, Risk

## Abstract

**Background:**

Use of antibiotic prophylaxis (AP) prior to invasive dental procedures (IDPs) to prevent infective endocarditis (IE) fell dramatically in the UK when the National Institute for Health and Care Excellence (NICE) recommended its cessation in 2008. However, guidance implementation by dentists, general medical practitioners (GPs) and others differed in England and Scotland, particularly after 2018 when the Scottish Dental Clinical Effectiveness Programme advised Scottish dentists to consider AP for high-risk individuals. We compared the effects of this differing guidance on AP prescribing and IE-admissions.

**Methods:**

A quasi-experimental (natural) longitudinal-study design was used to compare aggregated national AP prescribing (2002–2025) and IE-admissions data (1998–2025) for England and Scotland. Interrupted time series and difference-in-differences analyses were used to evaluate changes.

**Findings:**

AP prescribing fell 93% in England after the 2008 NICE guidelines and remained low (2567 [95% CI 2433–2701] prescriptions/million/annum before 2008 vs 185 [95% CI 165–204] prescriptions/million/annum after 2021/2, p < 0.0001), while IE-admissions increased by 112% (26 [95% CI 25–27] admissions/million/annum before 2008 vs 55 [95% CI 54–56]/million/annum after 2021/2, p < 0.0001). In Scotland, AP prescribing fell less, because GP prescribing replaced some dental prescribing, then rose again after 2018 and finished 2.86 times higher than in England (1884 [95% CI 1816–1952] prescriptions/million before 2008 vs 528 [95% CI 350–705] prescriptions/million after 2021/2). Correspondingly, Scottish IE-admissions rose less (24 [95% CI 23–25] admissions/million before 2008 vs 34 [95% CI 32–37] admissions/million after 2021/2, p < 0.0001) than in England, and English IE-admissions/million ended 61% higher than in Scotland (p < 0.0001).

**Interpretation:**

Although our data do not establish causality, they suggest that cessation of AP may be followed by a significant increase in IE-admissions compared with policies recommending AP for high-risk individuals.

**Funding:**

None.

**Research in context**

**Evidence before this study**

We searched PubMed for time-trend studies on the effect of antibiotic prophylaxis (AP) guideline change on infective endocarditis (IE) incidence using search terms; *infective endocarditis, antibiotic prophylaxis, guideline, and dental procedure* from inception until December 2025. We identified 22 studies of which 19 included pre-post statistical analysis; 9 with no significant change in IE-incidence, 7 with an increase and 3 a decrease. All hypothesised that recommendations reducing AP would result in increased IE-incidence if AP was effective and no change if it was ineffective. Most studies, however, were conducted in countries where guidelines continued to recommended AP for high-risk individuals, potentially hiding any effect. Others failed to quantify the effect of guidance change on AP prescribing, thus any lack of effect on IE-incidence could represent lack of compliance rather than AP ineffectiveness. Because IE is rare, and it takes time for guideline change to affect IE incidence, large populations and prolonged post change data collection are required to achieve the power to detect any statistically significant change. Few studies achieved this and none compared the implementation of guidance change in different nations or the effect of recommending AP reintroduction.

**Added value of this study**

This study examined the effect of recommending against AP and later recommending its reintroduction for high-risk patients on AP prescribing and IE-incidence. It is also the first to compare the implementation of such recommendations in two nations.

The UK National Institute for Health and Care Excellence recommended against AP for IE prevention in 2008, but this was implemented differently in England and Scotland. In England, rigorous implementation resulted in a fall in AP prescribing and an increase in IE admissions. Although NICE guidelines do not apply in Scotland, dentists were initially advised to follow them. However, overall AP prescribing in Scotland did not decline as markedly, because of continued physician prescribing. In 2018, the Scottish Dental Clinical Effectiveness Programme recommended dentists consider AP for high-risk individuals in Scotland. Overall AP prescribing in Scotland increased and is now 2.86 times higher than in England (528 vs 185 prescriptions/million/annum), and IE admissions (initially equivalent in England and Scotland [26 vs 25/million/annum]) are now 61% higher in England than in Scotland (55 vs 34/million/annum).

**Implications of all the available evidence**

Although our data do not establish causality, they suggest that cessation of AP may have contributed to the significant increase in IE admissions seen in England.

## Introduction

Infective endocarditis (IE) is uncommon, but has high morbidity and 30% first year mortality.^1^ Oral bacteria are implicated as causal organisms in 35–45% of cases,^2^, ^3^, ^4^, ^5^ and antibiotic prophylaxis (AP) prior to invasive dental procedures (IDPs) has been the focus of IE prevention for 70 years and remains the standard of care for ‘high-risk’ patients worldwide.^6^, ^7^

Before 2006, IE prevention guidelines in the UK were provided by the British Society for Antimicrobial Chemotherapy (BSAC) who recommended AP before IDPs for patients at high or moderate risk of IE, consistent with guidelines elsewhere at that time (Fig. 1). In 2006, however, BSAC updated this guidance and became the first to recommend restriction of AP to high-risk individuals (those with previous history of IE, prosthetic heart valves or valves repaired with prosthetic material, unrepaired cyanotic congenital heart disease, repaired congenital heart defects [for 6 months after surgery or lifelong if a palliative shunt, conduit or prosthesis remains], or a ventricular assist device) undergoing IDPs only.^8^ This provoked opposition,^8^, ^9^, ^10^ resulting in suspension of the BSAC guidance and the National Institute for Health and Care Excellence (NICE) was directed to undertake a review of IE prevention guidelines. When NICE Clinical Guidelines CG64 was published in April 2008, it surprised many with its conclusion that, *“antibiotic prophylaxis against infective endocarditis is not recommended for people undergoing dental procedures*”.^11^ The reasons given were (a) the lack of any randomised controlled trial (RCT) data demonstrating the efficacy of AP (a requirement for NICE guidance at that time); (b) concerns regarding the risk of associated adverse drug reactions; and (c) the outcome of a health economic analysis they performed. The health economic analysis was based on an assumption that AP was not effective (due to the lack of RCT evidence) and a relatively high estimate of the risk of adverse drug reactions.^12^, ^13^ It concluded AP was not cost-effective and should not be recommended.^14^

**Fig. 1:**
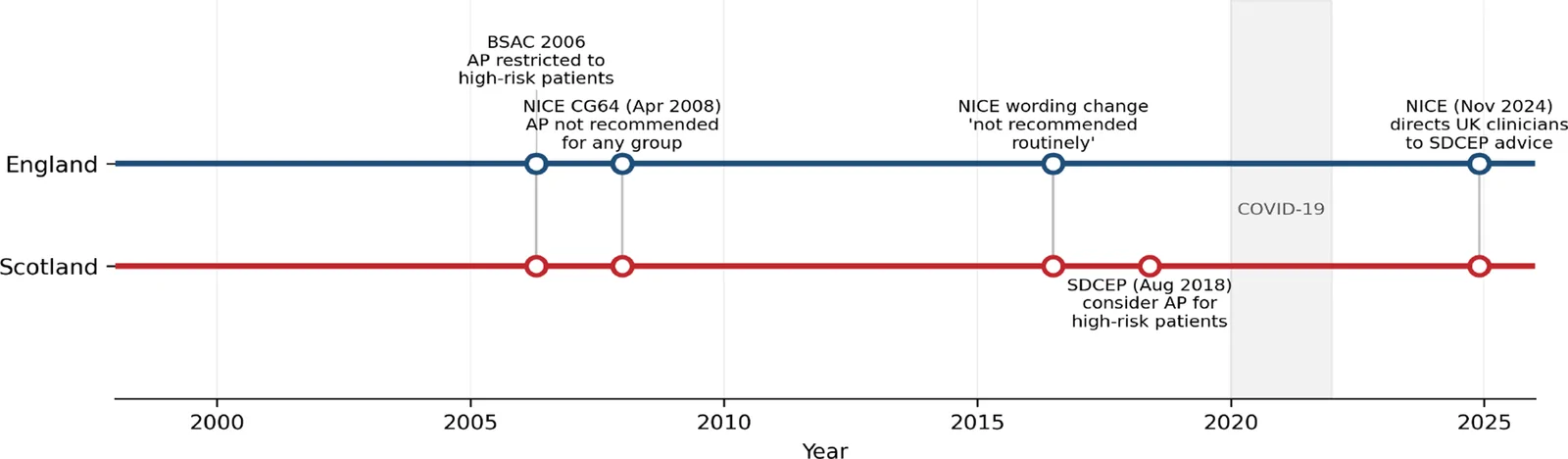
Timeline of infective endocarditis prophylaxis guidance in England and Scotland.

The UK was isolated in recommending against AP, whilst guidelines from the American Heart Association (AHA) in 2007,^15^ and European Society for Cardiology (ESC) in 2009,^16^ recommended that all high-risk individuals receive AP before any IDP, just as BSAC had recommended in 2006.^8^

Data demonstrating a significant increase in IE-admissions following the 2008 NICE guideline change^17^ prompted NICE to review its guidelines in 2015 but, in the absence of RCT data, it decided no change was needed. Nonetheless, in 2016, NICE changed the wording of its guidance without any further consultation or review,^18^ to read: *“Antibiotic prophylaxis against infective endocarditis is not recommended* ***routinely*** *for people undergoing dental procedures”*. The addition of the word ‘routinely’ caused confusion by implying that there were non-routine situations when AP might be appropriate, particularly since NICE provided no additional information as to when AP might be appropriate or which antibiotic regimen should be used.

NICE guidelines do not apply in Scotland, nonetheless, in 2008 the Chief Dental Officer for Scotland advised Scottish dentists to follow the NICE guidance even though they were not contractually required to do so (unlike their counterparts in England and Wales). However, this advice did not apply to general medical practitioners (GPs) or other healthcare workers in Scotland where many were therefore free to follow the ESC guidance recommending AP for high-risk individuals undergoing at-risk dental procedures.^6^, ^16^, ^19^

After NICE changed the wording of its guidance in 2016, the Scottish Dental Clinical Effectiveness Program (SDCEP) issued new advice in 2018 on how dentists in Scotland should implement the NICE guidance. This advice was based on the 2015 ESC guidelines, and recommended AP should be considered for high-risk individuals undergoing any at-risk dental procedures.^19^ Although SDCEP claimed NICE endorsement of their advice, this was not clearly stated within the NICE guidelines and most dentists in England and Wales continued to follow NICE guidance against the use of AP. It was only in November 2024 that NICE amended the wording of CG64 to indicate that clinicians across the UK (not just in Scotland) should follow the SDCEP advice (Fig. 1).

In this study, we therefore aimed to compare the impact of the differing AP guidance in Scotland and England on AP prescribing by dentists, GPs and other prescribers, and on IE-admissions.

## Methods

### Antibiotic prophylaxis prescribing

The recommended AP regimen prior to the 2008 NICE guidelines was a single 3 g oral dose of amoxicillin (or a 600 mg oral dose of clindamycin in penicillin-allergic individuals) and the 2018 SDCEP advice recommended the same. The dose, timing and mode of administration of amoxicillin and clindamycin are almost uniquely associated with AP prescribing for IDPs in the UK.^17^ Using the Freedom of Information Act, aggregated data on prescriptions for a single 3 g dose of oral amoxicillin or a single 600 mg dose of oral clindamycin were obtained from the National Health Service Business Services Authority in England and Public Health Scotland for the period January 2002 to March 2025. The prescribing data for this period was complete and included all community prescriptions (including hospital outpatient clinics) but did not encompass hospital inpatient use. Detailed prescribing data were unavailable before 2002.

### Infective endocarditis admissions

Using data from NHS England and Public Health Scotland (via the Freedom of Information Act), we identified all patients admitted to hospital (including those who died in hospital) with a primary ICD-10 discharge diagnosis of “acute or subacute infectious endocarditis” (ICD-10 code I33.0) between April 1998 and March 2025. The ICD-10 I33.0 IE-admissions data for this period was complete. We used ICD-10 code I33.0 to identify IE cases since this has high specificity (0.95) for the detection of Duke-positive IE cases (PPV 0.82, NPV 0.75), albeit with comparatively low sensitivity (0.45). The addition of other possible ICD-10 codes for IE (I38 and I39) has been shown to only marginally improve sensitivity (0.56) and NPV (0.78) but at the cost of significantly reduced specificity (0.90) and PPV (0.76).^20^

### Study design

This was a quasi-experimental (natural experiment) study of two national populations, using a controlled interrupted time series design with difference-in-differences estimation, and is reported in accordance with the STROBE statement for observational studies (Supplementary Material). Ethical approval and informed consent were not required for this study since it used de-identified, aggregate national data.

The unit of analysis was the financial year (April to March), labelled according to starting year (i.e. 2008 denotes 2008/09). Two outcomes were pre-specified: (i) the annual number of IE-hospital admissions, and (ii) the annual number of AP prescriptions in each nation. Both national datasets were complete.

Data concerning the type of AP prescription (all, 3 g amoxicillin or 600 mg clindamycin) and prescriber (dentist, GP, other [including nurse practitioners, pharmacists etc]) were also obtained (Supplementary Material).

### Model structure

Annual counts were modelled by negative binomial regression (NB2) with a log-link and the mid-year national population entered as a log offset, so that all parameters describe rates per head of population per annum (reported as incidence rate ratios [IRRs] with 95% confidence intervals). The negative binomial distribution was preferred to Poisson because of substantial overdispersion (Poisson Pearson dispersion statistic 1.4–7.0 for IE-admissions, 118–1356 for prescriptions). Details of the segmented regression model used, timing of interruptions, controlled interrupted time series analysis and difference-in differences analyses are provided in the Supplementary Methods.

### Autocorrelation

Autocorrelation was assessed using several different methods (see Supplementary Methods and Supplementary Table S9), none of which affected any primary estimate: two-period difference-in-differences estimates, and pre-intervention parallel-trends tests reached the same conclusion. While the method chosen did affect some secondary estimates, we explicitly state where a conclusion was sensitive to the choice (rather than reporting only the most favourable).

### Counterfactual estimation

To express the association in absolute terms, the number of English IE-admissions that would have been expected between 2008/09 and 2024/25 had the pre-2008 trend continued was estimated by setting all intervention terms to zero while retaining the COVID-19 indicator, followed by calculation of the cumulative difference from the observed count. Uncertainty was obtained by Monte Carlo sampling (20,000 draws) of the model coefficients from their estimated sampling distribution. These are not causal estimates but projections under a modelled counterfactual.

### Sensitivity analyses

Six sensitivity analyses were performed: excluding the COVID-19 years (2020/21 and 2021/22) for each outcome (rather than adjusting for them); fitting the prescribing model without an implementation period; restricting the IE series to 2002/03 onwards so that both outcomes covered identical periods; and refitting the controlled interrupted time series models for each outcome with Poisson rather than negative binomial errors. The conclusions concerning the reported model-based intervals were unchanged in every case (Supplementary Methods).

Analyses were performed in R (version 4.5.1) using the MASS package.

### Role of the funding source

There was no funding source for this study.

## Results

AP prescribing and IE-admissions data are provided in Table 1 and Figs. 2 and 3. The Supplementary Tables and Figures also provide further information regarding prescribing personnel (dentists, GPs, others) and the mode of AP prescribed. Segmented interrupted time series data are listed in Table 2 and Figs. 4 and 5, and difference-in-differences estimates comparing Scotland and England are included in Table 3.

**Table 1:** Antibiotic prophylaxis (AP) and infective endocarditis (IE) admissions per million population in England and Scotland.

Year	England			Scotland		
	Population	AP prescriptions/million	IE-admissions/million	Population	AP prescriptions/million	IE-admissions/million
1998–99	48,820,583		26.61	5,077,070		25.21
1999–00	49,032,872		28.63	5,071,950		22.67
2000–01	49,233,311		24.60	5,062,940		23.90
2001–02	49,449,746		25.30	5,064,200		23.89
2002–03	49,679,267	2611.93	25.66	5,066,000	1793.53	22.90
2003–04	49,925,517	2640.91	24.04	5,068,500	1832.10	23.28
2004–05	50,194,600	2595.76	25.20	5,084,300	1880.10	24.39
2005–06	50,606,034	2661.48	26.97	5,110,200	1903.45	23.48
2006–07	50,965,186	2576.29	24.39	5,133,000	1917.98	27.86
2007–08	51,381,093	2313.96	27.34	5,170,000	1977.18	23.40
2008–09	51,815,853	1395.54	27.60	5,202,900	1102.85	27.10
2009–10	52,196,381	574.66	26.88	5,231,900	425.08	21.60
2010–11	52,642,452	464.42	27.66	5,262,200	335.79	25.08
2011–12	53,107,169	365.00	33.97	5,299,900	326.61	19.62
2012–13	53,506,812	293.70	32.71	5,308,500	341.55	23.55
2013–14	53,918,686	269.07	37.02	5,317,300	326.14	27.27
2014–15	54,370,319	244.64	40.17	5,332,200	317.74	27.19
2015–16	54,808,676	226.33	44.57	5,351,700	305.01	27.09
2016–17	55,289,034	212.83	44.49	5,374,900	329.20	31.44
2017–18	55,619,548	195.36	46.49	5,389,900	300.47	37.11
2018–19	55,924,528	195.44	50.07	5,394,300	310.45	35.41
2019–20	56,230,056	208.32	51.38	5,414,400	410.81	29.55
2020–21	56,325,961	146.20	43.02	5,413,100	303.57	28.63
2021–22	56,554,891	185.31	53.58	5,418,400	380.39	29.34
2022–23	57,112,542	181.45	54.75	5,447,700	526.90	37.45
2023–24	57,690,323	171.06	56.70	5,490,100	553.58	34.97
2024–25	58,620,101	200.78	55.05	5,546,900	650.09	34.79

**Fig. 2:**
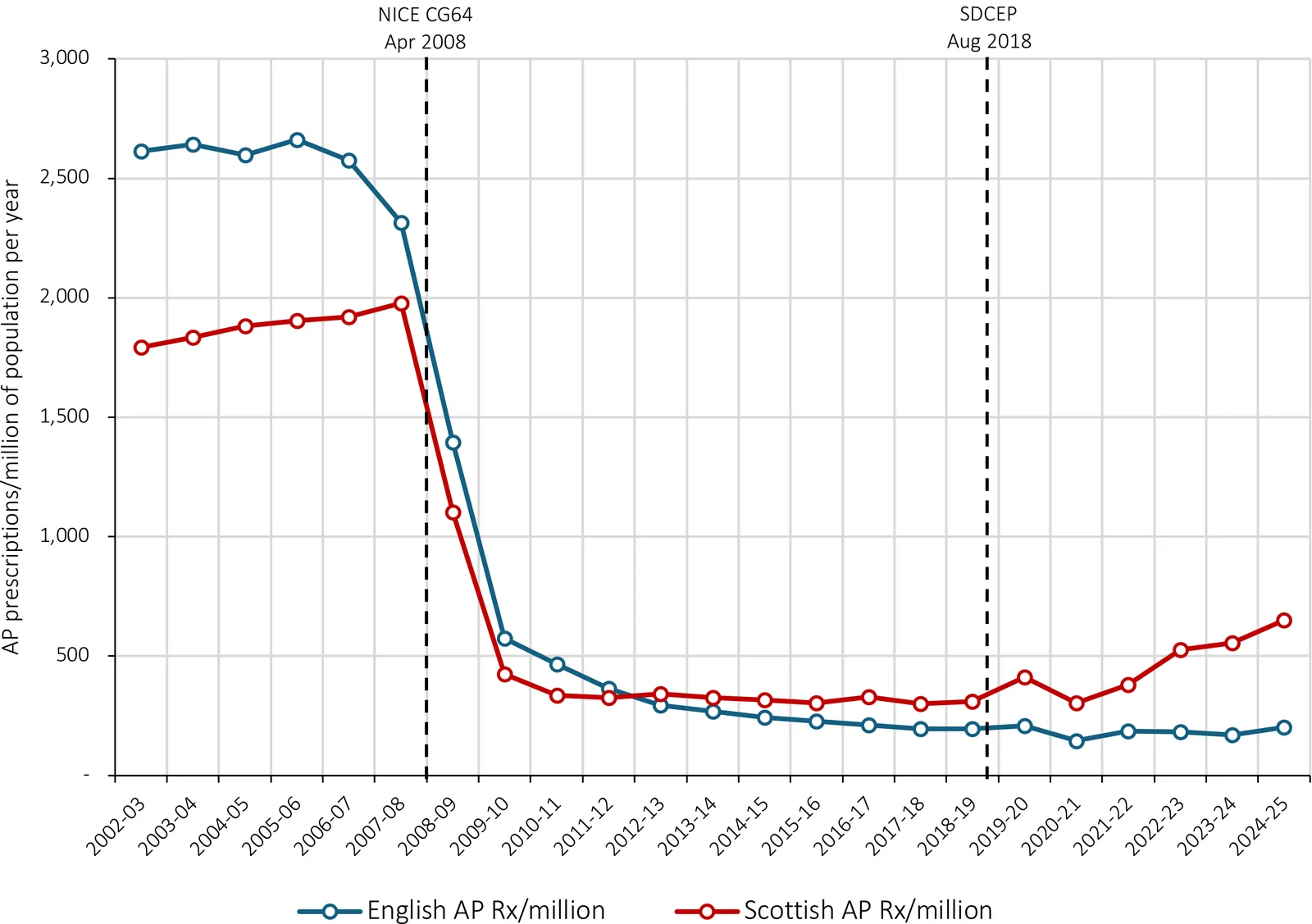
Antibiotic prophylaxis (AP) prescriptions (Rx)/million/annum in England and Scotland. NICE, National Institute for Health and Care Excellence guidance; SDCEP, Scottish Dental Clinical Effectiveness Programme implementation advice for the NICE guidance; AP, antibiotic prophylaxis; Rx, prescribing.

**Fig. 3:**
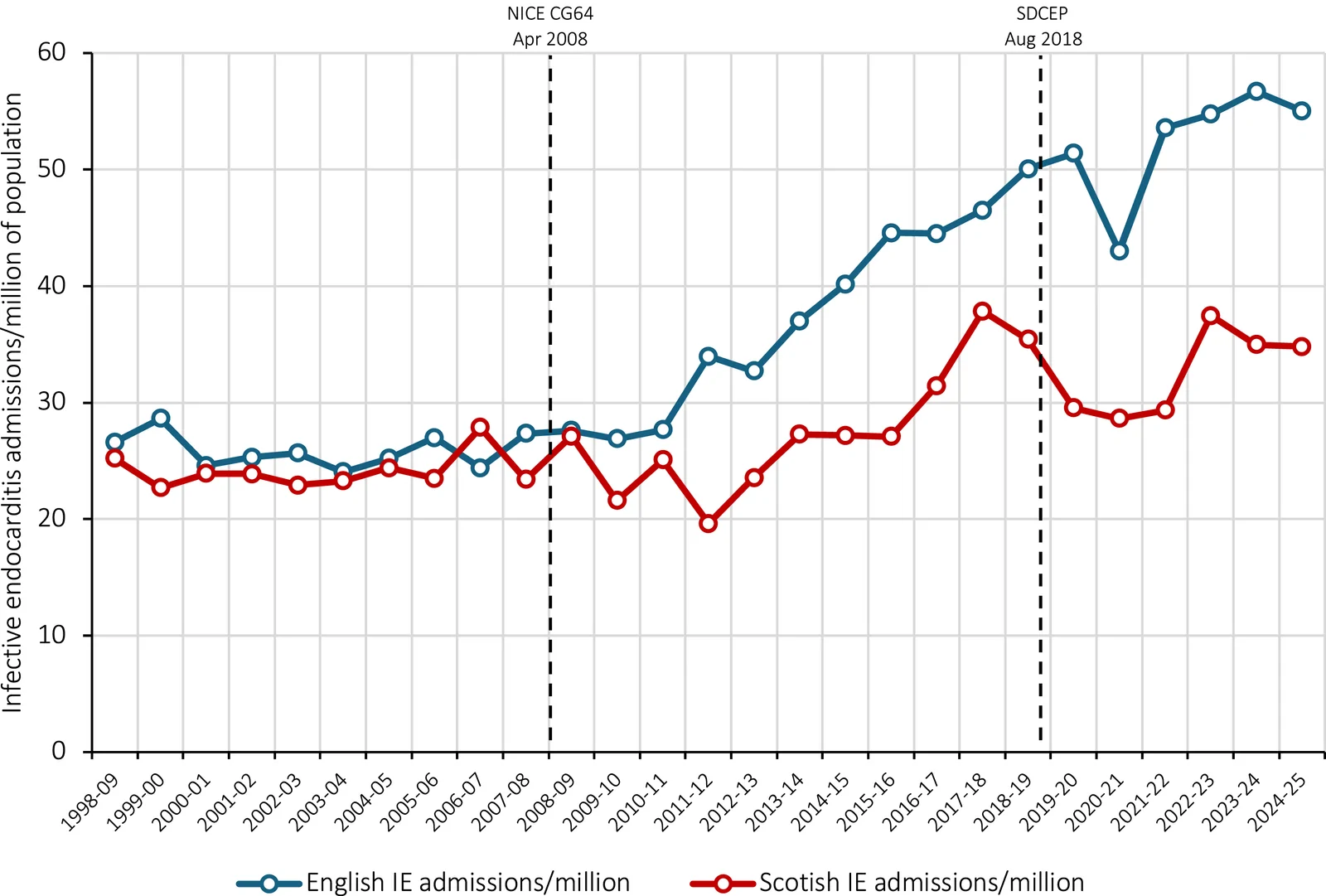
Infective endocarditis (IE) hospital admissions/million/annum in England and Scotland. NICE, National Institute for Health and Care Excellence guidance; SDCEP, Scottish Dental Clinical Effectiveness Programme implementation advice for the NICE guidance.

**Fig. 4:**
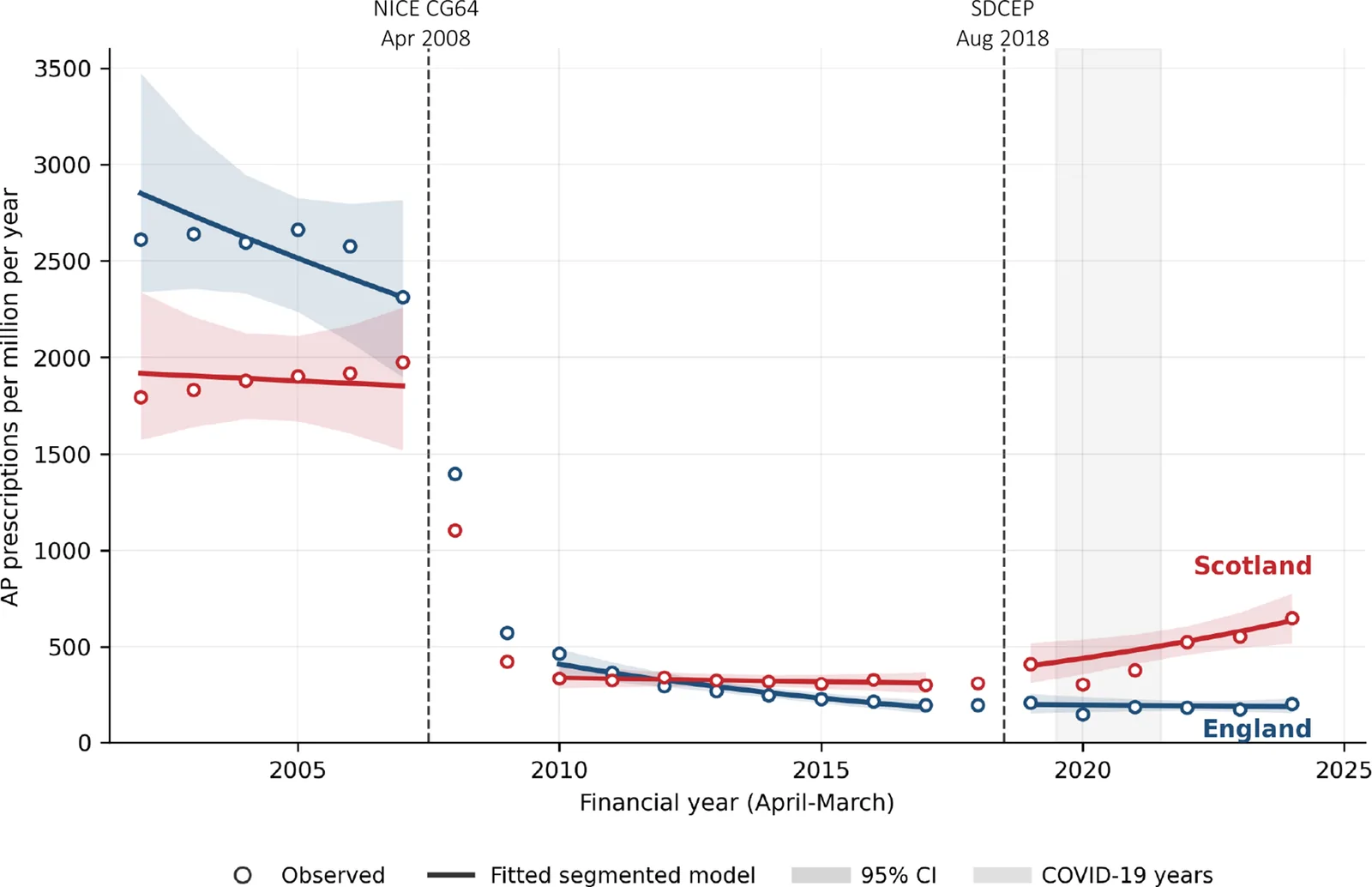
Segmented interrupted time series of antibiotic prophylaxis (AP) prescribing in England and Scotland. NOTE: Points are observed annual rates; lines are fitted segments from the negative binomial model with 95% confidence bands. Dashed vertical lines mark publication of NICE CG64 (April 2008) and the SDCEP implementation advice (August 2018); the grey band marks the COVID-19 years, which are adjusted for in the model but omitted from the plotted trend. The partially exposed 2018/19 year is shown as an observed point but is not joined by a fitted segment.

**Table 2:** Segmented interrupted time series estimates for antibiotic prophylaxis (AP) prescribing and infective endocarditis (IE) admissions.

Estimate	IRR	95% CI	p
**AP prescriptions—England**			
Pre-NICE annual trend	0.959	0.899–1.023	0.206
Post-NICE annual trend	0.891	0.854–0.930	<0.0001
Post-SDCEP annual trend	0.990	0.921–1.064	0.782
NICE level change	0.201	0.133–0.304	<0.0001
NICE slope change	0.929	0.860–1.004	0.063
Level change at 2019/20	1.352	0.942–1.941	0.102
Slope change from 2019/20	1.111	1.021–1.208	0.014
**AP prescriptions—Scotland**			
Pre-NICE annual trend	0.993	0.930–1.060	0.837
Post-NICE annual trend	0.987	0.945–1.031	0.554
Post-SDCEP annual trend	1.096	1.019–1.179	0.014
NICE level change	0.186	0.123–0.282	<0.0001
NICE slope change	0.994	0.919–1.075	0.876
Level change at 2019/20	1.337	0.927–1.928	0.121
Slope change from 2019/20	1.110	1.020–1.209	0.016
**IE-admissions—England**			
Pre-NICE annual trend	0.997	0.986–1.008	0.610
Post-NICE annual trend	1.070	1.059–1.082	<0.0001
Post-SDCEP annual trend	1.024	0.999–1.050	0.065
NICE level change	1.027	0.936–1.126	0.577
NICE slope change	1.073	1.057–1.091	<0.0001
Level change at 2019/20	0.918	0.817–1.033	0.155
Slope change from 2019/20	0.957	0.931–0.983	0.001
**IE-admissions—Scotland**			
Pre-NICE annual trend	1.005	0.986–1.025	0.615
Post-NICE annual trend	1.046	1.027–1.065	<0.0001
Post-SDCEP annual trend	1.034	0.995–1.075	0.092
NICE level change	0.874	0.746–1.023	0.094
NICE slope change	1.041	1.013–1.069	0.003
Level change at 2019/20	0.871	0.722–1.049	0.145
Slope change from 2019/20	0.988	0.947–1.032	0.593

Incidence rate ratios (IRR) with 95% confidence intervals (CI) from negative binomial models with log population offset.

**Fig. 5:**
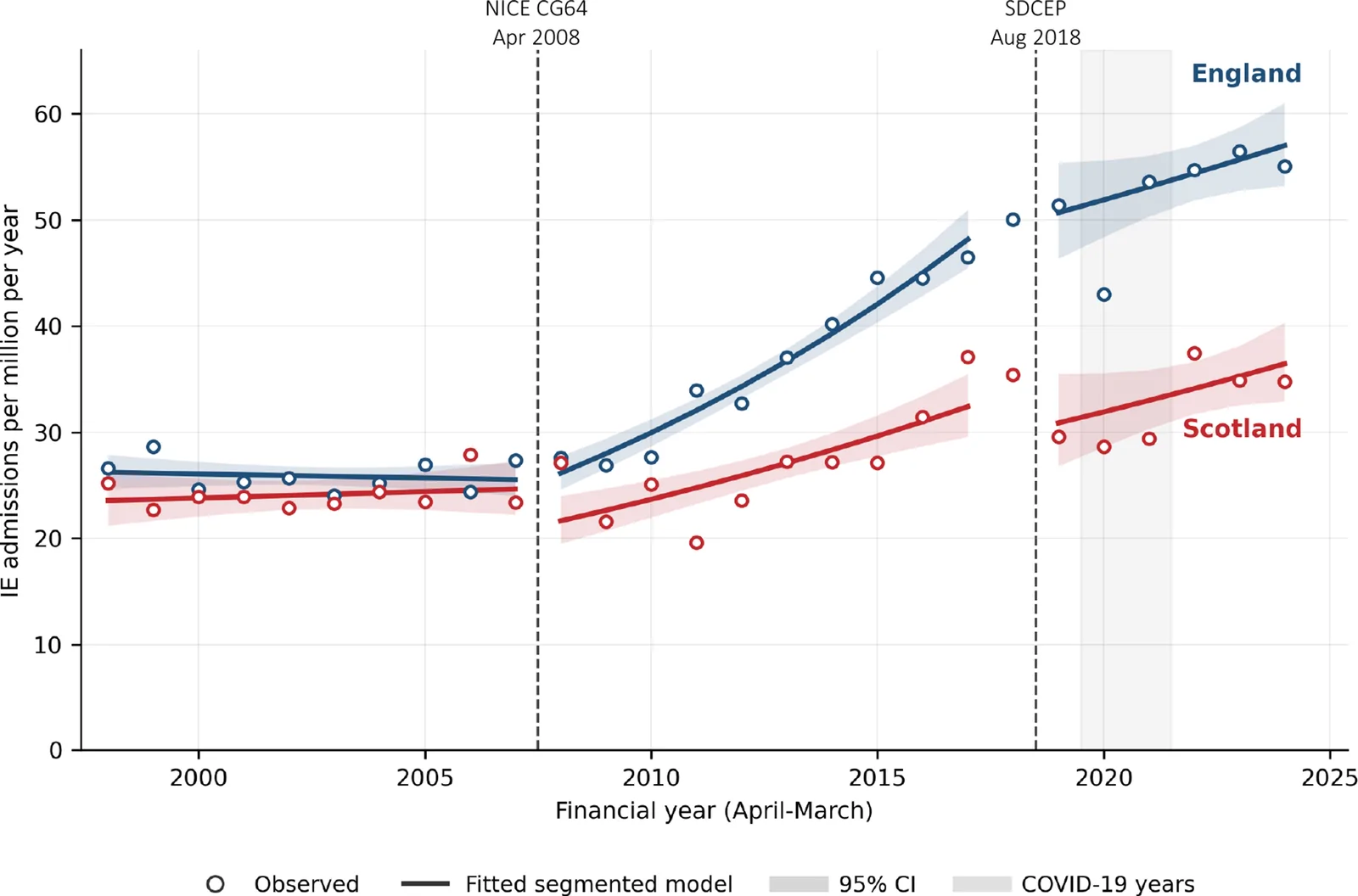
Segmented interrupted time series of infective endocarditis (IE) hospital admissions in England and Scotland. NOTE: Points are observed annual rates; lines are fitted segments from the negative binomial model with 95% confidence bands. Dashed vertical lines mark publication of NICE CG64 (April 2008) and the SDCEP implementation advice (August 2018); the grey band marks the COVID-19 years, which are adjusted for in the model but omitted from the plotted trend. The partially exposed 2018/19 year is shown as an observed point but is not joined by a fitted segment.

**Table 3:** Difference-in-differences estimates comparing Scotland with England over stable four-year windows.

Estimate	Ratio	95% CI	p
**AP prescriptions, NICE era (2004/05–07/08 vs 2014/15–17/18)**			
Observed rates, England		2536.0 → 219.6 per million/yr	
Observed rates, Scotland		1919.9 → 313.1 per million/yr	
England post vs pre (RR)	0.087	0.080–0.093	<0.0001
Scotland post vs pre (RR)	0.163	0.151–0.176	<0.0001
DiD (ratio of rate ratios)	1.883	1.692–2.095	<0.0001
Scotland vs England, pre-window	0.757	0.703–0.815	<0.0001
Scotland vs England, post-window	1.425	1.319–1.539	<0.0001
Parallel-trends test, AP prescribing (country × time interaction, pre-2008)	1.036	0.945–1.135	0.46
**AP prescriptions, SDCEP era (2014/15–17/18 vs 2021/22–24/25)**			
Observed rates, England		219.6 → 184.7 per million/yr	
Observed rates, Scotland		313.1 → 528.6 per million/yr	
England post vs pre (RR)	0.840	0.723–0.976	0.022
Scotland post vs pre (RR)	1.686	1.448–1.962	<0.0001
DiD (ratio of rate ratios)	2.006	1.621–2.483	<0.0001
Scotland vs England, pre-window	1.425	1.225–1.657	<0.0001
Scotland vs England, post-window	2.858	2.459–3.322	<0.0001
**IE-admissions, NICE era (2004/05–07/08 vs 2014/15–17/18)**			
Observed rates, England		26.0 → 44.0 per million/yr	
Observed rates, Scotland		24.8 → 30.7 per million/yr	
England post vs pre (RR)	1.691	1.569–1.823	<0.0001
Scotland post vs pre (RR)	1.240	1.085–1.417	0.002
DiD (ratio of rate ratios)	0.733	0.629–0.855	<0.0001
Scotland vs England, pre-window	0.954	0.852–1.068	0.415
Scotland vs England, post-window	0.699	0.630–0.776	<0.0001
Parallel-trends test, IE admissions (country × time interaction, pre-2008)	1.008	0.984–1.033	0.52
**IE-admissions, SDCEP era (2014/15–17/18 vs 2021/22–24/25)**			
Observed rates, England		44.0 → 55.0 per million/yr	
Observed rates, Scotland		30.7 → 34.1 per million/yr	
England post vs pre (RR)	1.251	1.178–1.328	<0.0001
Scotland post vs pre (RR)	1.111	0.988–1.249	0.080
DiD (ratio of rate ratios)	0.888	0.778–1.013	0.078
Scotland vs England, pre-window	0.699	0.636–0.769	<0.0001
Scotland vs England, post-window	0.621	0.567–0.680	<0.0001

Values are ratios of rate ratios; a value <1 indicates a smaller change in Scotland than in England, and a value >1 a larger change in Scotland than England.

### The effect of the 2008 NICE guidance

Before 2008, AP prescribing was stable in both countries and higher in England than in Scotland (2004/05–2007/08: 2567 vs 1884 prescriptions per million per year; rate ratio for Scotland vs England 0.76, 95% CI 0.70–0.81) (Table 3, Fig. 4). Following NICE CG64, prescribing fell steeply in both countries over the two-year implementation period and then stabilised at a much lower level.

This fall was substantially larger in England. Comparing 2004/05–2007/08 with 2014/15–2017/18 (Table 3), prescribing fell by 91.3% in England (rate ratio 0.087, 95% CI 0.080–0.093) and by 83.7% in Scotland (0.163, 95% CI 0.151–0.176), with a difference-in-differences of 1.88 (95% CI 1.69–2.09). In other words, Scottish prescribing ended the period 88% higher relative to its own baseline than in England. This estimate was obtained by comparing AP prescribing data for the four-year window immediately preceding the NICE guideline change (2004/05–2007/08) and immediately before publication of the SDCEP advice (2014/15–2017/18). However, the estimate was insensitive to the choice of pre-intervention window used in the analysis, ranging from 1.79 to 2.06 across every window possibility (different length and timing of windows before the NICE guidance [see Supplementary Table S8]).

English prescribing continued to decline after 2010/11 (10.9% per year, IRR 0.891, 95% CI 0.854–0.930, Table 2) whereas Scottish prescribing remained stable (1.3% per year, IRR 0.987, 95% CI 0.945–1.031) with a difference between these post-NICE trends of 1.11 per year (95% CI 1.04–1.18). By 2014/15–2017/18, Scottish prescribing *per capita* exceeded that in England (rate ratio 1.42, 95% CI 1.32–1.54, Table 3).

Notably, dental prescribing of AP after the 2008 NICE guidance fell in England and Scotland by the same amount (92%) (Supplementary Fig. S3), with a difference-in-differences of 0.98 (95% CI 0.82–1.18). However, overall AP prescribing fell less in Scotland than in England because, after an initial fall, GP prescribing of AP in Scotland increased by 16% after NICE CG64, while GP prescribing of AP fell by 86% in England (difference-in differences 8.31, 95% CI 6.94–9.94).

### The effect of the 2018 SDCEP advice

The SDCEP advice was followed by a change in prescribing in Scotland alone, where prescribing rose by 69% between 2014/15–2017/18 and 2021/22–2024/25 (313–528 per million; rate ratio 1.69, 95% CI 1.45–1.96, Table 3), while it continued to fall by 16% in England (220–185 per million; rate ratio 0.84, 95% CI 0.72–0.98, Table 3). The difference-in-differences was 2.01 (95% CI 1.62–2.48), and Scottish prescribing was 2.86 times higher than in England by 2021/22–2024/25 (95% CI 2.46–3.32). In the segmented model (Table 2, Fig. 4), Scottish prescribing changed from a flat trajectory before SDCEP to an increase of 9.6% per year thereafter (IRR 1.096, 95% CI 1.019–1.179), whilst English prescribing remained flat (IRR 0.990, 95% CI 0.921–1.064). The difference between the two post-2019 trends was 1.11 per year (95% CI 1.00–1.23).

### Changes in infective endocarditis admissions

#### The effect of the 2008 NICE guidance

Before 2008, IE admission rates were stable and similar in the two countries (Table 1, Figs. 3 and 5) (1998/99–2007/08 annual trend: England IRR 0.997, 95% CI 0.986–1.008; Scotland IRR 1.005, 95% CI 0.986–1.025, Table 2) and these two national trends were statistically indistinguishable (difference-in-differences for pre-NICE trends 1.008, 95% CI 0.984–1.032, Table 3), supporting the parallel-trends assumption underlying subsequent comparisons (see also Supplementary Methods and Supplementary Tables S5 and S6).

There was no abrupt change in the level of IE-admissions when the NICE guidance was introduced, but there was a marked change in trend in both countries. Thus, the annual trend in England changed from −0.3% per year before 2008 to +7.0% per year thereafter (post-NICE trend IRR 1.070, 95% CI 1.059–1.082; change in trend IRR 1.073, 95% CI 1.057–1.091, Table 2). The corresponding change was smaller in Scotland: from +0.5% to +4.6% per year (post-NICE trend IRR 1.046, 95% CI 1.027–1.065; change in trend IRR 1.041, 95% CI 1.013–1.069).

Comparing 2004/05–2007/08 with 2014/15–2017/18 (Table 3), IE-admissions rose by 69% in England (26.0–44.0 per million; rate ratio 1.69, 95% CI 1.57–1.82) and by 24% in Scotland (24.8–30.7 per million; rate ratio 1.24, 95% CI 1.08–1.42). The difference-in-differences was 0.73 (95% CI 0.63–0.85), indicating that the increase in IE-admissions in Scotland was 27% smaller than that in England over the period of divergent prescribing. Like the AP prescribing data, this estimate was obtained by comparing IE-admissions for the four-year window immediately preceding the NICE guideline change (2004/05–2007/08) and immediately before publication of the SDCEP advice (2014/15–2017/18). This estimate was insensitive to the choice of pre-intervention window, ranging from 0.71 to 0.76 across every window from the 2–10 years preceding the guidance (Supplementary Table S8).

Had the pre-2008 English trend continued, an estimated 23,032 IE-admissions would have occurred between 2008/09 and 2024/25 against the 40,279 observed, a cumulative excess of 17,247 admissions (95% CI 13,848–20,164, Supplementary Fig. S4). In Scotland, the equivalent estimate was 351 excess admissions (95% CI −47 to 685).

### The effect of the 2018 SDCEP advice

IE admission rates continued to diverge, and the rate in England was 54.96 per million per year (95% CI 54.00–55.92) by 2021/22–2024/25 compared with 34.14 per million per year in Scotland (95% CI 31.69–36.58), an absolute difference of 20.8 admissions per million per year (95% CI 18.2–23.5; rate ratio of 1.61, 95% CI 1.50–1.73). However, the majority of this divergence had accrued before the SDCEP advice was published.

## Discussion

Introduction of the NICE guidelines recommending cessation of all AP prescribing to prevent IE in 2008 was associated with a significant fall in AP prescribing in England followed by a significant increase in IE-admissions. In contrast, there was a smaller (but still statistically significant) fall in AP prescribing in Scotland, partly because the fall in AP prescribing by Scottish dentists (which was proportionately similar to that in England) was compensated by continued GP prescribing of AP in Scotland (Supplementary Fig. S3). Indeed, Scottish GPs prescribed AP more frequently than dentists after 2015/16, reflecting their adherence to ESC guidance recommending AP for high-risk patients.

The advice from SDCEP in August 2018 that AP should be considered for high-risk individuals resulted in a significant increase in AP prescribing by Scottish dentists, GPs, and other prescribers, but (perhaps understandably) had no impact in England. Consequently, mean AP prescribing in Scotland between 2021/22 and 2024/25 was 2.86 times higher than for the same period in England. Moreover, the data showed that AP prescribing continued to increase in Scotland during that period (from 380.39 prescriptions/million in 2021/2022 to 650.09 in 2024/25) whilst remaining comparatively flat in England (185.31 prescriptions/million in 2021/22 to 200.78 in 2024/25).

Prior to 2008, IE-admissions per million of population were stable and statistically indistinguishable in England and Scotland (26 vs 25 IE-admissions/million). Since then, IE-admissions have increased steadily in England (to 55 admissions/million), but less so in Scotland (to 34 admissions/million), meaning that IE-admissions in England are now 61% higher than in Scotland.

The key question is whether the fall in AP prescribing that followed the introduction of the NICE guidelines in 2008 was responsible for the subsequent increase in IE-admissions. Although the strong temporal association does not prove a causal relationship, pre-2008 trends in the two countries were statistically indistinguishable, whilst the trends changed in both countries after 2008 such that the country where prescribing fell less (Scotland) had the smaller rise in IE-admissions (difference-in-differences of 0.73; 95% CI 0.63–0.85).

The second question is whether the 2018 SDCEP advice changed AP prescribing in both countries? The fact that AP prescribing rose 69% after 2018 in Scotland, whilst English prescribing fell by 16% (difference-in-differences 2.01; 95% CI 1.62–2.48), suggests that the SDCEP advice only impacted on AP prescribing in Scotland, probably because dentists in England were either unaware of the SDCEP advice or thought it applied to practitioners in Scotland only.

The flat trajectory of IE-admissions in Scotland after 2018 suggests that increased AP prescribing in Scotland could have helped reduce IE-admissions. However, IE-admission trends in England also slowed significantly from 2019/20 (IRR 0.957, 95% CI 0.932–0.982), despite a lack of exposure to the SDCEP advice. Indeed, when the part-exposed year 2019/20 is treated as pandemic-affected, or excluded, the two countries slow by almost the same amount (England 0.946, 95% CI 0.901–0.993; Scotland 0.937, 95% CI 0.857–1.023) and the difference between them disappears (0.990, 95% CI 0.895–1.095). The same shared prescribing discontinuity is present in both countries (Supplementary Table S5) and a UK-wide influence around 2019–2020—changes in coding or referral practice, onset of the COVID-19 pandemic, or slowing of the underlying rise—is therefore likely to be contributing to both national series. This common influence is largely removed by the between-country contrast on which our inferences rest, but it is a further reason to regard the SDCEP-era estimates as inconclusive rather than confirmatory. Indeed, our analyses suggest that the majority of the divergence in IE-admissions between England and Scotland accrued over the decade following the NICE guidelines rather than after the 2018 SDCEP advice. The lag between changing use of AP and any effect on IE-admissions (and the fact that we are only able to report 6-years of post-SDCEP data) means that further years of follow up will be needed to confirm whether the SDCEP advice itself significantly reduced IE incidence.

Cessation of AP use in those at moderate- and high-risk of IE in 2008 was a major shift in practice and AP prescribing in England fell by 93% as a consequence. Notably, it did not fall to zero and questionnaire data suggest that this residual prescribing was primarily targeted at high-risk individuals.^21^ The impact of the NICE guidelines was less dramatic in Scotland, where AP prescribing was already lower than in England and fell much less before stabilising swiftly (whilst English prescribing continued to fall). Indeed, AP prescribing *per capita* exceeded that in England from 2012 onwards and subsequently increased in response to the SDCEP implementation advice. The current annual rate of 530 AP prescriptions/million of population in Scotland remains lower than that before the 2008 NICE guidance but is likely to be targeted at high-risk individuals. In contrast, prescribing rates remain substantially lower in England (185/million/annum), providing a potential explanation for the higher number of current IE-admissions compared to Scotland (55/million/annum vs 34/million/annum), whereas there was no statistically significant difference in IE-admissions between the two nations before 2008. The present difference in IE-admissions (21/million/annum), suggests that IE-admissions could hypothetically be reduced by 1231, cases per annum in England if AP prescribing levels were the same as in Scotland. Furthermore, with an associated one-year mortality of ∼30%, this change could hypothetically be associated with an annual reduction of 6.3 IE deaths per million of population (approximately 369 IE deaths per annum in England).

Accruing research evidence, including large population-based studies demonstrating that AP significantly reduces IE incidence following invasive dental procedures in those at high-risk,^22^, ^23^ led the ESC to increase the strength of their AP recommendation from Class IIa to Class I (the highest).^6^ Since then further evidence of AP effectiveness has accrued including a systematic review and meta-analysis.^24^, ^25^ These developments led to a call, in this journal in April 2024,^12^ for NICE to review its guidelines. Soon after, NICE initiated a surveillance review that concluded in October 2024 that a full review was unnecessary. Nonetheless, in the context of all this, NICE further amended the wording of its guidance in November 2024. It now reads, *“Antibiotic prophylaxis against infective endocarditis is not recommended* ***routinely*** *for people undergoing dental procedures. For advice on antibiotic prophylaxis for people at high risk of infective endocarditis undergoing dental procedures and for relevant patient information, see the Scottish Dental Clinical Effectiveness Programme's implementation advice on antibiotic prophylaxis against infective endocarditis (this programme is part of NHS Education for Scotland). It has been endorsed for use across the UK by the Chief Dental Officers of the UK”*.^26^

For the first time NICE has acknowledged the existence of individuals at high risk for IE and that they might benefit from AP. Additionally, NICE is now directing clinicians across the UK (not just in Scotland) to follow the SDCEP implementation advice. Furthermore, SDCEP updated its implementation advice in April 2026 to align more closely with the 2023 ESC recommendations.^27^ These sequential changes mean that AP is now recommended in the UK for high-risk individuals undergoing extractions or oral surgical procedures, and should be considered for all other invasive dental procedures involving manipulation of the gingival or periapical region of the teeth. After 18 years when AP was not recommended, only time will determine if this abrupt change in IE prevention guidance will result in a reduction in IE hospital admissions in England, Wales and Northern Ireland as already observed in Scotland.

There are several limitations to this study. The IE-admissions data rely upon UK hospital coding generated by trained and accredited coders but may not be generalisable to other populations. Although this coding is subject to error, other studies have shown that it provides reliable and complete data capture for UK hospital admissions.^28^ Furthermore, coding was undertaken independently of our study and was therefore not subject to bias or influenced by changes to the guidelines. Moreover, the size and scale of the data set (and the consistency of the underlying coding process) are likely to negate the impact of any systematic error. Although IE may present to a variety of hospital specialities, causing difficulties in initial diagnosis, hospital episode statistics record the final diagnosis for each episode and accurately reflect the true number of IE cases. Nonetheless, the diagnosis of IE is sometimes uncertain and may not always be based upon standardised criteria.^29^, ^30^

Although this study focussed on the differences in AP prescribing between England and Scotland and their impact on IE-admissions, other differences between the nations could have contributed to our observations. Potential confounders considered included population demographics, prevalence of patients with prosthetic (surgical or transcatheter) valves and cardiac implantable electronic devices (CIEDs), haemodialysis, intravenous drug use (IVDU), diabetes and improvements in diagnostic imaging. The Scottish population was slightly older (by ∼2 years) than that in England and remained so over the period of the study (increasing from a median age of 37.0 in 2002 to 40.5 in 2025 in England [an increase of 3.5 years] and from 38.8 to 42.3 years [an increase of 3.5 years] in Scotland), possibly making the Scottish population minimally more susceptible to IE. The proportion of men was minimally higher in England than Scotland and did not change significantly over the period of the study (increasing from 48.8% to 49.0% in England and from 48.5% to 48.6% in Scotland), likely countering any age disparity between the nations for IE-risk. Rates of valve replacement were similar in both nations, and the proportion of transcatheter valve interventions increased in parallel. CIED implantation rates and use of haemodialysis were lower in Scotland, whilst IVDU has been higher. Scotland's burden of drug-related harm rose steeply over the period of this study and remains the highest in the United Kingdom, with drug-misuse deaths rising to a peak 2.8 times that of England and Northern Ireland in 2023. If drug-use-associated IE followed that gradient, it would have added disproportionately to Scottish rather than English admissions and biased the England-vs-Scotland difference-in-differences towards the null. The divergence we report is thus unlikely to be an artefact of differential injecting-drug use. Conversely, the prevalence of diabetes and access to diagnostic imaging is slightly lower in Scotland. Although it is impossible to discount the effect of these confounders completely, their effects are likely to have been small and relatively balanced between the two study populations.

The available data bear some asymmetry in that IE-admissions data were available for 10-years prior to the 2018 NICE guidelines (1998/99–2007/08) whilst AP prescribing data was only available for 6-years (2002/03–2007/08). This asymmetry is evident in the data tables and time course plots and may have affected some interrupted time series analyses (Table 2). However, it should not have affected the difference-in-differences analyses where the same pre-NICE guideline four-year window (2004/05–2007/08) was used for IE-admissions and AP prescribing.

Finally, confining IE-admissions to those caused by oral bacterial species would have identified those admissions most likely to have been associated with changes in AP, but data concerning causative pathogens were unavailable.

### Conclusions

Differing implementation of NICE guidelines in England and Scotland between 2008 and 2024/5 has resulted in differing levels of AP prescribing that could explain observed differences in the incidence of IE-admissions between the two nations. These data suggest that use of AP for high-risk individuals undergoing invasive dental procedures may reduce the overall incidence of IE-admissions. Recent updates in the UK guidelines are therefore timely, and AP is now recommended for high-risk individuals undergoing extractions and oral surgery procedures and should be considered for all other at-risk procedures involving manipulation of the gingival or periapical region of teeth. Continued evaluation is needed to determine whether AP prescribing in England, Wales and Northern Ireland will increase because of this change and whether this will reduce the overall number of IE-admissions (as seems to be the case in Scotland).

## Contributors

All authors contributed to the study design, writing of the paper and the decision to publish. Prescribing and IE-admissions data were gathered, analysed and vouched for by MT and MD. MT and MD had full access to and verified all the data.

## Data sharing statement

This study used publicly available national data obtained under the Freedom of Information Act from NHS Digital (https://digital.nhs.uk), the NHS Business Services Authority (NHSBSA; https://www.nhsbsa.nhs.uk/prescription-data) and Public Health Scotland (https://publichealthscotland.scot/resources-and-tools/health-intelligence-and-data-management) and Office for National Statistics (https://www.ons.gov.uk).

## Declaration of interests

We have read and understood the policy on declaration of interests and declare the following.

MT reports no competing interests.

BP reports receipt of speaker fees from Edwards Lifesciences and Polares Medical, and expert testimony payments related to IE (but not in the last 3 years). He also reports being Chair of Data Safety Monitoring Committee (Valvosoft) and Partner and Board member of CERC CRO.

MD reports no competing interests.

PL reports no competing interests.

LB reports royalty payments for authorship duties from UpToDate, Inc.
