# Supplementary material for: Changes in antibiotic prophylaxis prescribing and infective endocarditis admissions in England and Scotland following changes in national guidance: a quasi-experimental longitudinal study
Source: Lancet Reg Health Eur. 2026 Nov 7;72:101923. doi: 10.1016/j.lanepe.2026.101923 (PMC13763124; doi:10.1016/j.lanepe.2026.101923)
Supplement: Supplementary file 1 [file mmc1.pdf]

Guideline changes and their impact on antibiotic prophylaxis prescribing and endocarditis admissions in England and Scotland – Supplementary Materials

Martin H. Thornhill[1], Bernard Prendergast[2], Mark Dayer[3], Peter Lockhart[4], Larry M. Baddour[5]
[1]Emeritus Professor of Oral Medicine, School of Clinical Dentistry, University of Sheffield, Sheffield, UK. [2]Chair of Cardiology, Cleveland Clinic, London, UK. [3]Associate Professor and Consultant Cardiologist, Cardiovascular Research Institute, Mater Private Network, Dublin, Ireland, and Faculty of Health, University of Plymouth, Plymouth, UK. Research Professor, Department of Oral Medicine/Oral & Maxillofacial Surgery, Wake Forest University School of Medicine, Atrium Health – Carolinas Medical Center, Charlotte, NC, USA. UK. [6]Professor Emeritus, Division of Public Health, Infectious Diseases, and Occupational Health, Departments of Medicine and Cardiovascular Medicine, Mayo Clinic College of Medicine and Science, Rochester, Minnesota, USA.

Correspondence to:
Prof. Martin Thornhill, Tel: +44 (0)114-271-7857, Fax: +44 (0)114-271-7863, Email: m.thornhill@sheffield.ac.uk

Table of Contents: **Page**

Supplementary methods 2
Table S1. Prescribing of amoxicillin antibiotic prophylaxis in England 5
Table S2. Prescribing of clindamycin antibiotic prophylaxis in England 6
Figure S1(a). English prescribing of 3g amoxicillin AP/million of population 7
Figure S1(b). English prescribing of 600mg clindamycin AP/million of population 8
Figure S1(c). English AP prescribing/million of population by type of antibiotic 9
Figure S1(d). English AP prescribing/million of population by prescriber type 10
Table S3. Prescribing of amoxicillin antibiotic prophylaxis in Scotland 11
Table S4. Prescribing of clindamycin antibiotic prophylaxis in Scotland 12
Figure S2(a). Scottish prescribing of 3g amoxicillin AP/million of population 13
Figure S2(b). Scottish Prescribing of 600mg Clindamycin AP/million of Population 14
Figure S2(c). Scottish AP prescribing/million of population by type of antibiotic 15
Figure S2(d). Scottish AP prescribing/million of population by prescriber type 16
Figure S3. A comparison of AP prescribing by dentists and GPs in England and Scotland 17
Figure S4. Cumulative excess of IE admissions in England comparing observed admissions with continuation of the pre-NICE trend 18
Table S5. Controlled interrupted time series (segmented difference-in-differences) analysis 19
Table S6. Tests of the parallel trends assumption 21
Table S7. IE and AP annual trends in England and Wales immediately preceding the SDCEP advice 21
Table S8. Sensitivity of the NICE-era two-period difference-in-differences to the choice of pre-intervention window 22
Table S9 Residual autocorrelation diagnostics 24
STROBE Checklist 25

Supplementary Methods:

Study design and reporting

This was a quasi-experimental (natural experiment) study of two national populations, using a controlled interrupted time series design with difference-in-differences estimation. It is reported in accordance with the STROBE statement for observational studies; a completed STROBE checklist is provided supplementary material (page 22).

The unit of analysis was the financial year (April to March) labelled by their starting year (i.e. 2008 denotes 2008/09). Two outcomes were pre-specified: (i) the annual number of hospital admissions with a primary discharge diagnosis of ICD-10 I33.0, available for both countries for 1998/99 to 2024/25 (27 years per country, 54 observations); and (ii) the annual number of dispensed prescriptions for a single 3 g dose of oral amoxicillin or a single 600 mg dose of oral clindamycin, available for 2002/03 to 2024/25 (23 years per country, 46 observations). Both national datasets were complete, with no missing country-years within these periods.

Model structure

Annual counts were modelled by negative binomial regression (NB2) with a log-link and the mid-year national population entered as a log offset, so that all parameters describe rates per head of population and are reported as incidence rate ratios (IRRs) with 95% confidence intervals. The negative binomial distribution was preferred to Poisson because of substantial overdispersion (Poisson Pearson dispersion statistic 1.4 to 7.0 for IE admissions and 118 to 1356 for prescriptions); the Akaike information criterion favoured the negative binomial specification in every model except the Scottish IE series, for which the estimated overdispersion was negligible and the negative binomial model reduced to its Poisson limit.

For each country and outcome, a segmented regression of the following form was fitted:

$$\log E(Y\_t) = \log(\text{population}\_t) + \beta 0 + \beta 1\cdot \text{time}\_t + \beta 2\cdot \text{NICE}\_\text{level}\_t + \beta 3\cdot \text{NICE}\_\text{slope}\_t + \beta 4\cdot \text{SDCEP}\_\text{level}\_t + \beta 5\cdot \text{SDCEP}\_\text{slope}\_t + \beta 6\cdot \text{transition}\_t + \beta 7\cdot \text{COVID}\_t$$

where time is the number of years since the start of the series (so β1 is the underlying pre-intervention trend), NICE_level is an indicator taking the value 1 from the first post-NICE year onwards (so β2 is the immediate change in level at that point), and NICE_slope counts the years elapsed since that point, taking the value 0 in its first year (so β3 is the change in annual trend after NICE, and β1 + β3 is the post-NICE trend). SDCEP_level and SDCEP_slope are defined analogously for the second interruption.

Timing of the interruptions

NICE clinical guideline CG64 was published in April 2008, at the start of financial year 2008/09, which was therefore treated as the first exposed year. The SDCEP implementation advice was published in August 2018, part-way through 2018/19; that year was therefore only partially exposed, was given its own indicator (transition) and was excluded from the estimation of both the preceding and the following segments, with 2019/20 treated as the first fully exposed year. An indicator for 2020/21 and 2021/22 adjusted for disruption to dental services and hospital admissions during the COVID-19 pandemic.

AP prescribing did not decline log-linearly after 2008: it fell steeply over two years to a new, much lower plateau. Modelling this as a single log-linear post-intervention segment would have been a misspecification that propagated into the later estimates, forcing the model to fit a spurious upward step in 2019 in both countries in order to prevent the extrapolated exponential decline from falling below the observed plateau. For the prescribing outcome only, 2008/09 and 2009/10 were therefore modelled as an implementation period with their own indicator, and the post-NICE level and slope were estimated from 2010/11 onwards. This adjustment was made after inspecting the fit of the initial model rather than being specified in advance, and we report the unadjusted specification as a sensitivity analysis. No such adjustment was required for IE admissions, whose post-2008 trajectory was well described by a log-linear trend.

Controlled interrupted time series and difference-in-differences

To exploit the divergence between the two countries, the country series were then modelled jointly, with England as the reference and every term interacted with an indicator for Scotland. In this controlled ITS the interaction terms are difference-in-differences estimates: the country-by-time interaction over the pre-intervention period tests the parallel-trends assumption, and the interactions with the level and slope terms estimate the extent to which the Scottish response to each guideline change differed from the English response.

In this joint model the country-by-time interaction over the pre-intervention segment is a formal test of the parallel-trends assumption: it is a single coefficient, with its own standard error, whose exponent is the ratio of the Scottish to the English annual trend before the intervention, so that a value of 1 indicates parallel log-linear trends. It is reported in Table 3 and in Supplementary Tables S5 and S6, the latter re-estimating it in a model fitted to the pre-intervention window alone.

Segmented models assume a log-linear trend within each segment. Because the English prescribing series was a steep fall followed by a plateau rather than a constant proportional decline, we also pre-specified a conventional two-period difference-in-differences analysis that makes no assumption about the shape of the trajectory between the periods compared. Two stable four-year windows were compared using a negative binomial model with terms for country, period and their interaction, fitted to the individual country-year counts within each window (16 observations) rather than to a single collapsed total per country-period, so that year-to-year overdispersion is still estimated; the interaction is the ratio of rate ratios (RRR). For the SDCEP comparison the windows were 2014/15 to 2017/18 (after prescribing in both countries had stabilised following NICE, and before SDCEP) and 2021/22 to 2024/25 (after SDCEP, excluding the two COVID-affected years). For the NICE comparison the windows were 2004/05 to 2007/08 and 2014/15 to 2017/18. This two-period analysis was the primary test of whether SDCEP changed prescribing in Scotland alone. The post-intervention window was determined by the requirement that it begin after prescribing in both countries had stabilised following NICE and end before the SDCEP advice; the pre-intervention window was matched to it in length and placed immediately before the intervention. The same two windows were used for both outcomes so that the prescribing and admissions contrasts refer to identical periods. Prescribing data were available from 2002/03 and the two earliest years are included in the interrupted time series and in the test of parallel trends; the two-period contrast uses the four years immediately preceding the guidance. Supplementary Table S8 shows that the estimate is insensitive to this choice.

Autocorrelation and uncertainty

Residual autocorrelation was assessed using the Durbin-Watson statistic, the lag-1 autocorrelation of Pearson residuals, and Ljung-Box tests (see Table S9). In every model the lag-1 residual autocorrelation was negative rather than positive (−0.14 to −0.53; Durbin-Watson 2.24 to 3.01), indicating year-to-year oscillation rather than the positive serial dependence that ordinarily inflates apparent precision in interrupted time series. The Ljung-Box test was consistent with independence for the three IE models ($p=0.24$, 0.21 and 0.58 at lag 2) but rejected it for the three prescribing models ($p=0.019$, 0.121 and 0.004), which is what negative lag-1 correlation of this size produces.

All inference was therefore repeated using a Newey-West heteroskedasticity- and autocorrelation-consistent (HAC) covariance matrix with one lag, computed within country so that lags were never taken across the England-Scotland boundary. Across the 98 estimates reported, the HAC interval was narrower than the model-based interval in 75 and wider in 23 (width ratio 0.13 to 1.65, median 0.69); because the residual autocorrelation is negative, HAC narrows most intervals rather than widening them. The choice does not affect any primary estimate: the two-period difference-in-differences estimates and the pre-intervention parallel-trends tests reach the same conclusion under either variance estimator. It does affect some secondary estimates, and where a conclusion is sensitive to the choice we say so explicitly rather than reporting only the more favourable interval.

Counterfactual estimation

To express the association in absolute terms, the number of English IE admissions that would have been expected between 2008/09 and 2024/25 had the pre-2008 trend continued was estimated by setting all

intervention terms to zero while retaining the COVID-19 indicator, and the cumulative difference from the observed count was calculated. Uncertainty was obtained by Monte Carlo sampling (20,000 draws) of the model coefficients from their estimated sampling distribution. These are projections under a modelled counterfactual and are not causal estimates (see Table S4).

Sensitivity analyses

Six sensitivity analyses were performed: excluding the COVID-19 years (2020/21 and 2021/22) rather than adjusting for them, for each outcome; fitting the prescribing model without an implementation period; restricting the IE series to 2002/03 onwards so that both outcomes covered identical periods; and refitting the controlled interrupted time series models with Poisson rather than negative binomial errors, for each outcome. On the model-based intervals reported here, the conclusions were unchanged in every case. However, two qualifications are worth stating. Omitting the implementation period from the prescribing model widens the intervals substantially, as expected from a misspecified mean structure, although the point estimates are similar. Excluding rather than adjusting for the COVID-19 years leaves the segmented SDCEP slope interaction for IE admissions unchanged on the model-based interval (1.042, 95% CI 0.988 to 1.098) but moves it just past the conventional threshold on the HAC interval (95% CI 1.002 to 1.083); we do not regard this as evidence of a post-SDCEP divergence, for the reasons given in the Results.

Software and data availability

Analyses were performed in R (version 4.4 or later) using the MASS package.

Supplementary Material

Table S1· Prescribing of amoxicillin antibiotic prophylaxis in England

Year	Dent Amox AP	GP Amox AP	Other Amox AP	All Amox AP	Population Of England	Dent Amox AP /mil	GP Amox AP /mil	Other Amox AP /mil	All Amox AP /mil
2002-03	92,879	6,131	243	99,253	49,679,267	1,869·57	123·41	4·89	1,997·88
2003-04	94,201	6,302	251	100,754	49,925,517	1,886·83	126·23	5·03	2,018·09
2004-05	93,087	6,285	247	99,619	50,194,600	1,854·52	125·21	4·92	1,984·66
2005-06	96,472	6,169	233	102,874	50,606,000	1,906·34	121·90	4·60	2,032·84
2006-07	94,560	6,741	255	101,556	50,965,200	1,855·38	132·27	5·00	1,992·65
2007-08	84,431	5,867	212	90,510	51,381,100	1,643·23	114·19	4·13	1,761·54
2008-09	52,328	5,274	168	57,770	51,815,900	1,009·88	101·78	3·24	1,114·91
2009-10	22,530	2,814	105	25,449	52,196,400	431·64	53·91	2·01	487·56
2010-11	18,950	2,172	102	21,224	52,642,500	359·98	41·26	1·94	403·17
2011-12	14,868	1,831	68	16,767	53,107,200	279·96	34·48	1·28	315·72
2012-13	12,051	1,461	111	13,623	53,506,800	225·22	27·30	2·07	254·60
2013-14	11,312	1,306	82	12,699	53,918,700	209·79	24·21	1·52	235·52
2014-15	10,572	1,150	53	11,775	54,370,300	194·44	21·15	0·97	216·57
2015-16	9,854	1,003	43	10,900	54,808,700	179·79	18·30	0·78	198·87
2016-17	9,388	860	50	10,298	55,289,000	169·80	15·55	0·90	186·26
2017-18	8,537	846	44	9,427	55,619,500	153·49	15·21	0·79	169·49
2018-19	8,413	765	59	9,237	55,924,500	150·43	13·68	1·05	165·17
2019-20	8,969	839	80	9,888	56,230,100	159·51	14·92	1·42	175·85
2020-21	6,396	537	51	6,984	56,326,000	113·55	9·53	0·91	123·99
2021-22	7,882	727	75	8,684	56,554,900	139·37	12·85	1·33	153·55
2022-23	7,639	695	71	8,405	57,144,400	133·68	12·16	1·24	147·08
2023-24	7,130	734	87	7,951	57,932,500	123·07	12·67	1·50	137·25
2024-25	8,748	837	120	9,705	58,620,100	297·64	253·11	85·63	165.56

Annual prescribing (Rx) of a single 3g oral dose of amoxicillin (Amox) antibiotic prophylaxis (AP) in England by prescriber type. Dent = dentist, GP = general medical practitioner, Other = pharmacist, nurse practitioner or other prescriber, All = all AP prescriptions.

Table S2. Prescribing of clindamycin antibiotic prophylaxis in England

Year	Dent Clind AP	GP Clind AP	Other Clind AP	All Clind AP	Population Of England	Dent Clind AP /mil	GP Clind AP /mil	Other Clind AP /mil	All Clind AP /mil
2002-03	27,792	2,650	64	30,506	49,679,267	559·43	53·34	1·29	614·06
2003-04	28,027	2,997	71	31,095	49,925,517	561·38	60·03	1·42	622·83
2004-05	27,933	2,674	67	30,674	50,194,600	556·49	53·27	1·33	611·10
2005-06	28,678	3,063	72	31,813	50,606,000	566·69	60·53	1·42	628·64
2006-07	26,122	3,557	66	29,745	50,965,200	512·55	69·79	1·30	583·63
2007-08	24,578	3,712	94	28,384	51,381,100	478·35	72·24	1·83	552·42
2008-09	11,887	2,604	50	14,541	51,815,900	229·41	50·25	0·96	280·63
2009-10	3,319	1,203	24	4,546	52,196,400	63·59	23·05	0·46	87·09
2010-11	2,241	961	22	3,224	52,642,500	42·57	18·26	0·42	61·24
2011-12	1,813	786	18	2,617	53,107,200	34·14	14·80	0·34	49·28
2012-13	1,430	646	16	2,092	53,506,800	26·73	12·07	0·30	39·10
2013-14	1,216	577	17	1,809	53,918,700	22·55	10·69	0·31	33·55
2014-15	1,002	507	17	1,526	54,370,300	18·43	9·32	0·31	28·07
2015-16	1,017	477	11	1,505	54,808,700	18·56	8·70	0·20	27·46
2016-17	986	465	18	1,469	55,289,000	17·83	8·41	0·33	26·57
2017-18	998	422	19	1,439	55,619,500	17·94	7·59	0·34	25·87
2018-19	1,088	584	21	1,693	55,924,500	19·45	10·44	0·38	30·27
2019-20	1,229	571	26	1,826	56,230,100	21·86	10·15	0·46	32·47
2020-21	881	345	25	1,251	56,326,000	15·64	6·13	0·44	22·21
2021-22	1,320	443	33	1,796	56,554,900	23·34	7·83	0·58	31·76
2022-23	1,439	482	43	1,964	57,144,400	25·18	8·43	0·75	34·37
2023-24	1,419	494	46	1,959	57,932,500	24·49	8·53	0·79	33·82
2024-25	1,488	518	59	2,065	58,620,100	9·19	4·15	0·36	35·23

Annual prescribing (Rx) of a single 600mg oral dose of clindamycin (Clind) antibiotic prophylaxis (AP) in England by prescriber type. Dent = dentist, GP = general medical practitioner, Other = pharmacist, nurse practitioner or other prescriber, All = all AP prescriptions.

Figure S1(a) English prescribing of 3g amoxicillin AP/million of population

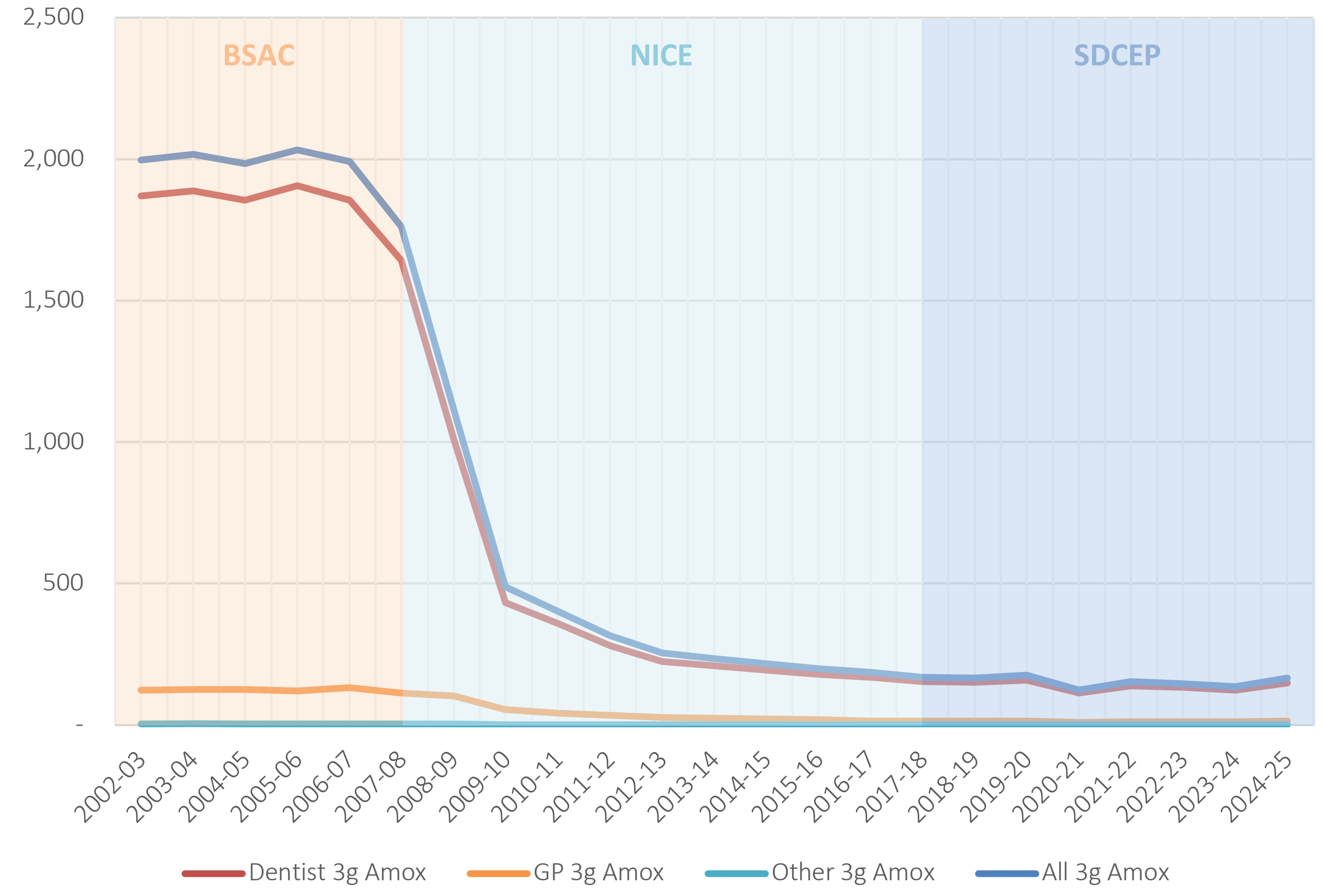

BSAC = British Society for Antimicrobial Chemotherapy guidance, NICE = National Institute for Health and Care Excellence guidance, SDCEP = Scottish Dental Clinical Effectiveness Programme implementation advice for the NICE guidelines, AP = antibiotic prophylaxis, Amox = amoxicillin AP, GP = general medical practitioner, Other = pharmacist, nurse practitioner or other prescriber

Figure S1(b). English prescribing of 600mg clindamycin AP/million of population

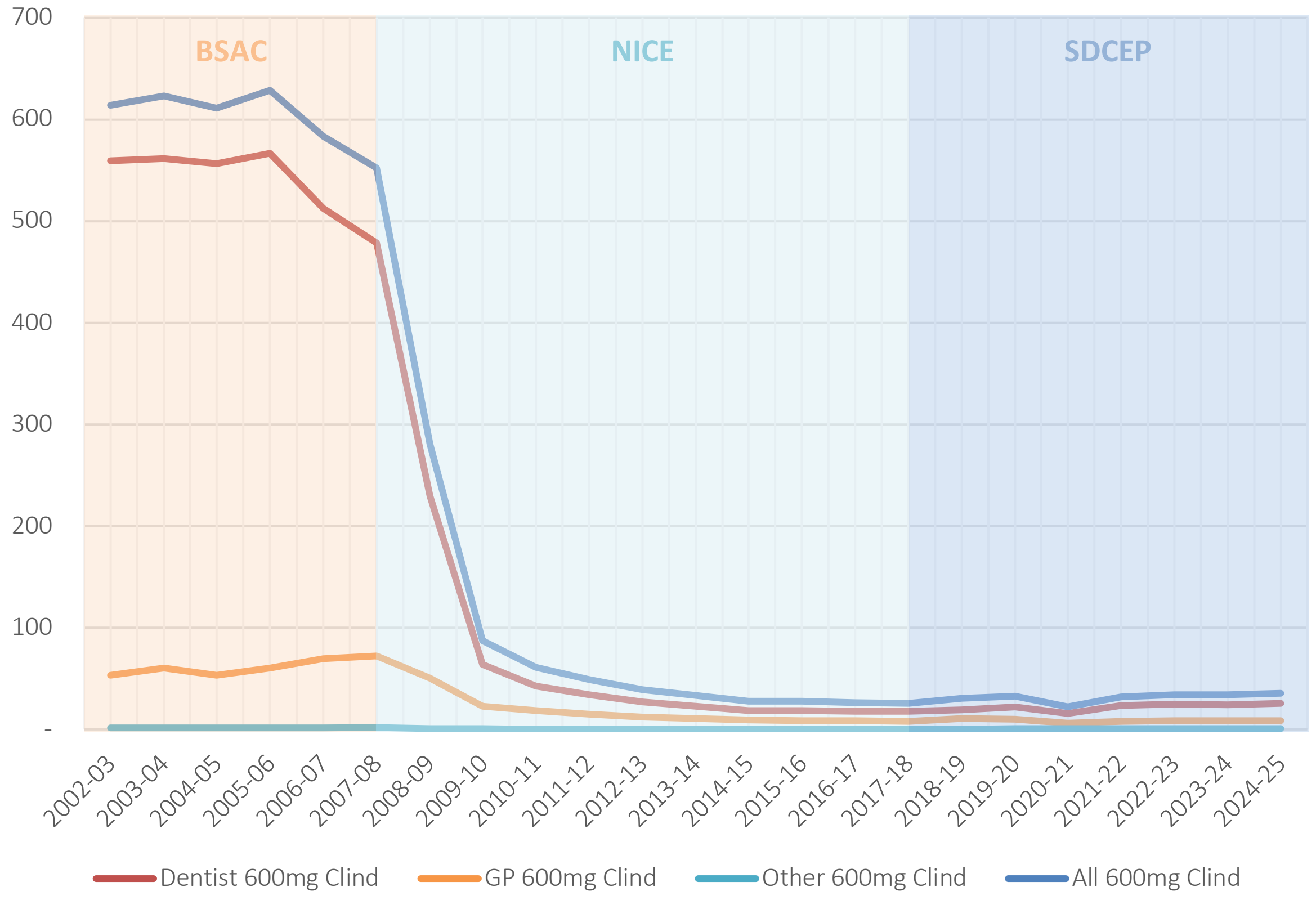

BSAC = British Society for Antimicrobial Chemotherapy guidance, NICE = National Institute for Health and Care Excellence guidance, SDCEP = Scottish Dental Clinical Effectiveness Programme implementation advice for the NICE guidelines, AP = antibiotic prophylaxis, Clind = clindamycin AP, GP = general medical practitioner, Other = pharmacist, nurse practitioner or other prescriber

Figure S1(c). English AP prescribing/million of population by type of antibiotic

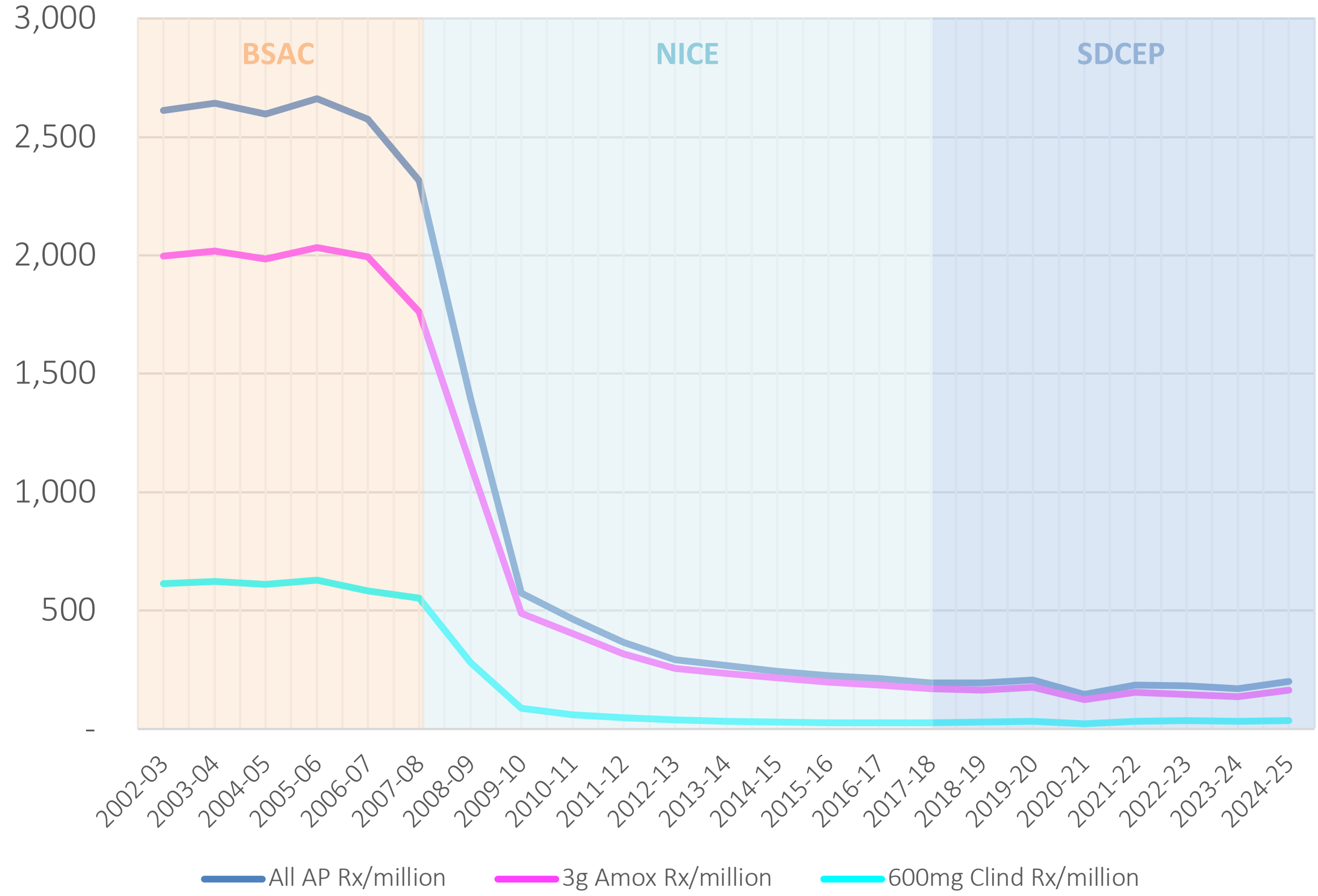

BSAC = British Society for Antimicrobial Chemotherapy guidance, NICE = National Institute for Health and Care Excellence guidance, SDCEP = Scottish Dental Clinical Effectiveness Programme implementation advice for the NICE guidelines, AP = antibiotic prophylaxis, Amox = amoxicillin AP, Clind = clindamycin AP

Figure S1(d). English AP prescribing/million of population by prescriber type

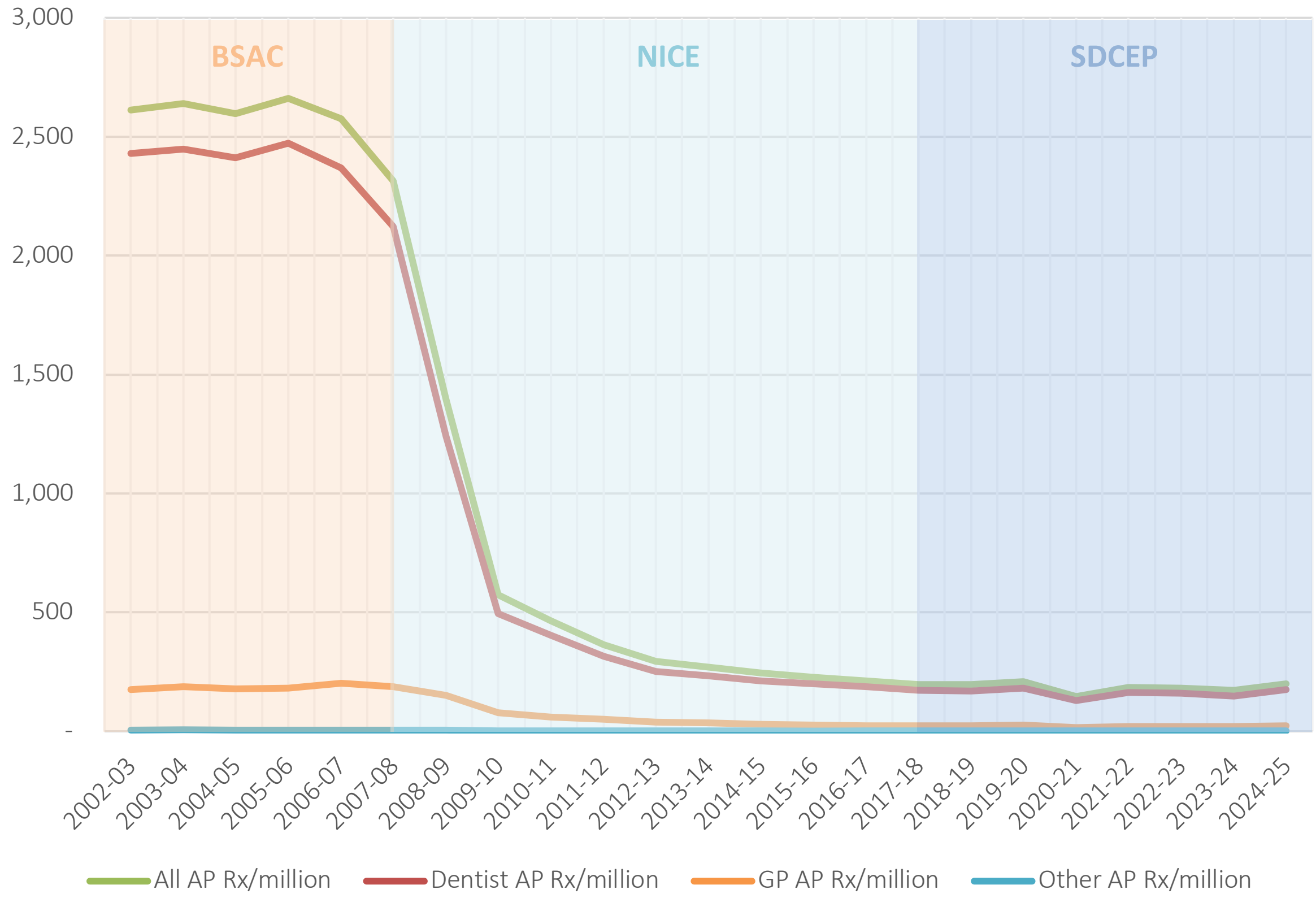

BSAC = British Society for Antimicrobial Chemotherapy guidance, NICE = National Institute for Health and Care Excellence guidance, SDCEP = Scottish Dental Clinical Effectiveness Programme implementation advice for the NICE guidelines, AP = antibiotic prophylaxis, GP = general medical practitioner, Other = pharmacist, nurse practitioner or other prescriber

Table S3. Prescribing of amoxicillin antibiotic prophylaxis in Scotland

Year	Dent Amox AP	GP Amox AP	Other Amox AP	All Amox AP	Population Of Scotland	Dent Amox AP /mil	GP Amox AP /mil	Other Amox AP /mil	All Amox AP /mil
2002-03	7,123	483	11	7,617	5,066,000	1,406·04	95·34	2·17	1,503·55
2003-04	7,233	523	13	7,769	5,068,500	1,427·05	103·19	2·56	1,532·80
2004-05	7,368	495	19	7,882	5,084,300	1,449·17	97·36	3·74	1,550·26
2005-06	7,430	503	16	7,949	5,110,200	1,453·95	98·43	3·13	1,555·52
2006-07	7,439	547	26	8,012	5,133,000	1,449·25	106·57	5·07	1,560·88
2007-08	7,756	578	24	8,358	5,170,000	1,500·19	111·80	4·64	1,616·63
2008-09	4,320	517	28	4,865	5,202,900	830·31	99·37	5·38	935·06
2009-10	1,629	401	26	2,056	5,231,900	311·36	76·65	4·97	392·97
2010-11	1,210	403	26	1,639	5,262,200	229·94	76·58	4·94	311·47
2011-12	1,146	470	20	1,636	5,299,900	216·23	88·68	3·77	308·69
2012-13	1,224	484	32	1,740	5,308,200	230·59	91·18	6·03	327·79
2013-14	1,053	602	18	1,673	5,316,800	198·05	113·23	3·39	314·66
2014-15	941	652	38	1,631	5,331,400	176·50	122·29	7·13	305·92
2015-16	734	785	54	1,573	5,350,600	137·18	146·71	10·09	293·99
2016-17	703	933	82	1,718	5,373,600	130·82	173·63	15·26	319·71
2017-18	567	866	108	1,541	5,388,200	105·23	160·72	20·04	286·00
2018-19	655	816	111	1,582	5,392,100	121·47	151·33	20·59	293·39
2019-20	1,011	927	176	2,114	5,411,200	186·83	171·31	32·53	390·67
2020-21	739	716	138	1,593	5,408,900	136·63	132·37	25·51	294·51
2021-22	965	870	160	1,995	5,412,900	178·28	160·73	29·56	368·56
2022-23	1,191	1,319	260	2,770	5,447,000	218·65	242·15	47·73	508·54
2023-24	1,296	1,325	344	2,965	5,506,000	235·38	240·65	62·48	538·50
2024-25	1,651	1,404	475	3,530	5,546,900	297·64	253·11	85·63	636·39

Annual prescribing (Rx) of a single 3g oral dose of amoxicillin (Amox) antibiotic prophylaxis (AP) in Scotland by prescriber type. Dent = dentist, GP = general medical practitioner, Other = pharmacist, nurse practitioner or other prescriber, All = all AP prescriptions.

Table S4. Prescribing of clindamycin antibiotic prophylaxis in Scotland

Year	Dent Clind AP	GP Clind AP	Other Clind AP	All Clind AP	Population Of Scotland	Dent Clind AP /mil	GP Clind AP /mil	Other Clind AP /mil	All Clind AP /mil
2002-03	1,368	98	3	1,469	5,066,000	270·04	19·34	0·59	289·97
2003-04	1,381	132	4	1,517	5,068,500	272·47	26·04	0·79	299·30
2004-05	1,536	139	2	1,677	5,084,300	302·11	27·34	0·39	329·84
2005-06	1,614	164	-	1,778	5,110,200	315·84	32·09	-	347·93
2006-07	1,662	164	7	1,833	5,133,000	323·79	31·95	1·36	357·10
2007-08	1,677	182	5	1,864	5,170,000	324·37	35·20	0·97	360·54
2008-09	790	82	1	873	5,202,900	151·84	15·76	0·19	167·79
2009-10	133	34	1	168	5,231,900	25·42	6·50	0·19	32·11
2010-11	91	33	4	128	5,262,200	17·29	6·27	0·76	24·32
2011-12	79	16	-	95	5,299,900	14·91	3·02	-	17·92
2012-13	53	20	-	73	5,308,200	9·98	3·77	-	13·75
2013-14	45	15	1	61	5,316,800	8·46	2·82	0·19	11·47
2014-15	35	27	1	63	5,331,400	6·56	5·06	0·19	11·82
2015-16	32	27	-	59	5,350,600	5·98	5·05	-	11·03
2016-17	24	27	-	51	5,373,600	4·47	5·02	-	9·49
2017-18	41	33	4	78	5,388,200	7·61	6·12	0·74	14·48
2018-19	41	45	6	92	5,392,100	7·60	8·35	1·11	17·06
2019-20	63	45	1	109	5,411,200	11·64	8·32	0·18	20·14
2020-21	27	22	-	49	5,408,900	4·99	4·07	-	9·06
2021-22	39	24	1	64	5,412,900	7·21	4·43	0·18	11·82
2022-23	66	30	4	100	5,447,000	12·12	5·51	0·73	18·36
2023-24	61	21	1	83	5,506,000	11·08	3·81	0·18	15·07
2024-25	51	23	2	76	5,546,900	9·19	4·15	0·36	13·70

Annual prescribing (Rx) of a single 600mg oral dose of clindamycin (Clind) antibiotic prophylaxis (AP) in Scotland by prescriber type. Dent = dentist, GP = general medical practitioner, Other = pharmacist, nurse practitioner or other prescriber, All = all AP prescriptions.

Figure S2(a) Scottish prescribing of 3g amoxicillin AP/million of population

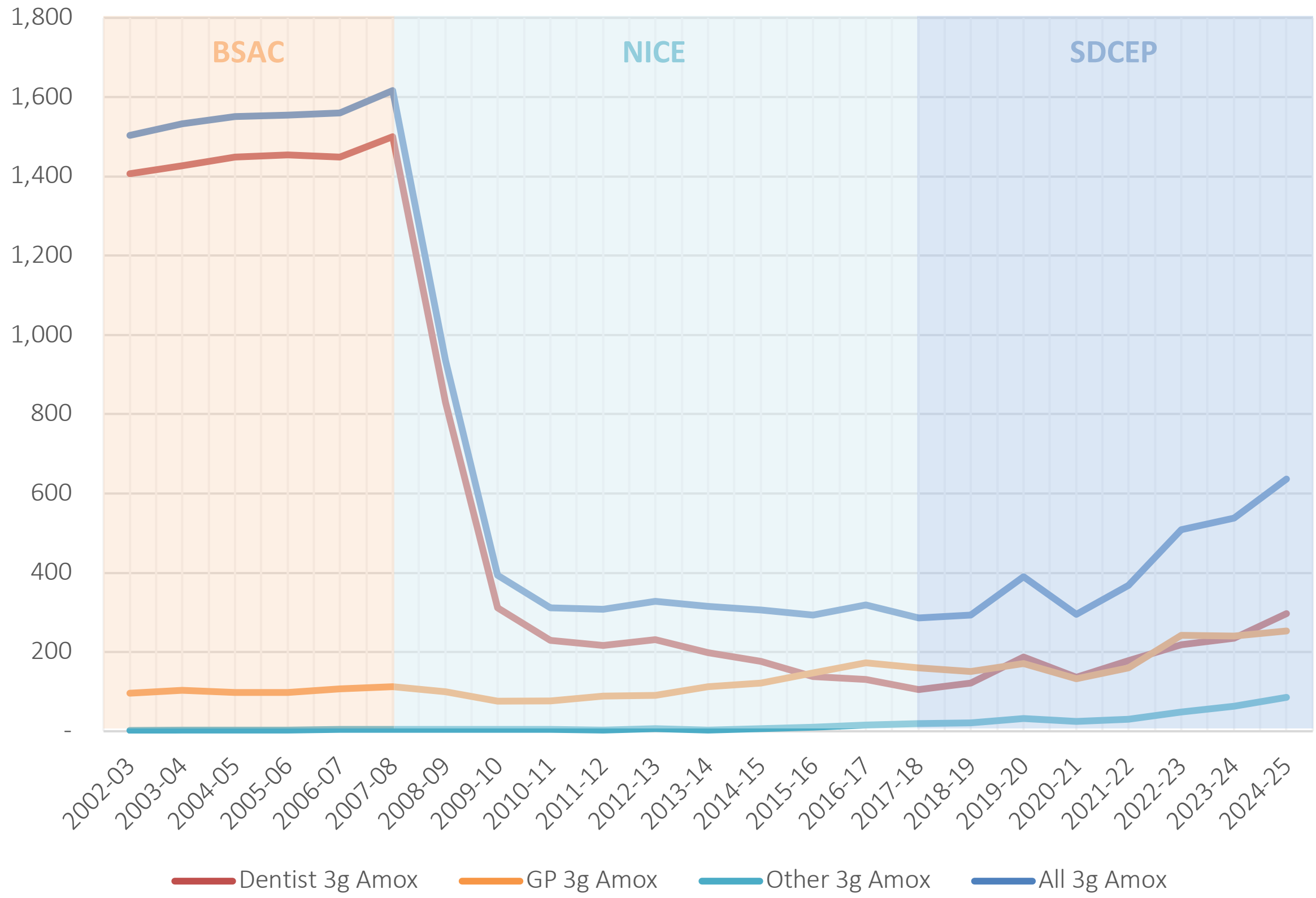

BSAC = British Society for Antimicrobial Chemotherapy guidance, NICE = National Institute for Health and Care Excellence guidance, SDCEP = Scottish Dental Clinical Effectiveness Programme implementation advice for the NICE guidelines, AP = antibiotic prophylaxis, Amox = amoxicillin AP, GP = general medical practitioner, Other = pharmacist, nurse practitioner or other prescriber

Figure S2(b). Scottish prescribing of 600mg clindamycin AP/million of population

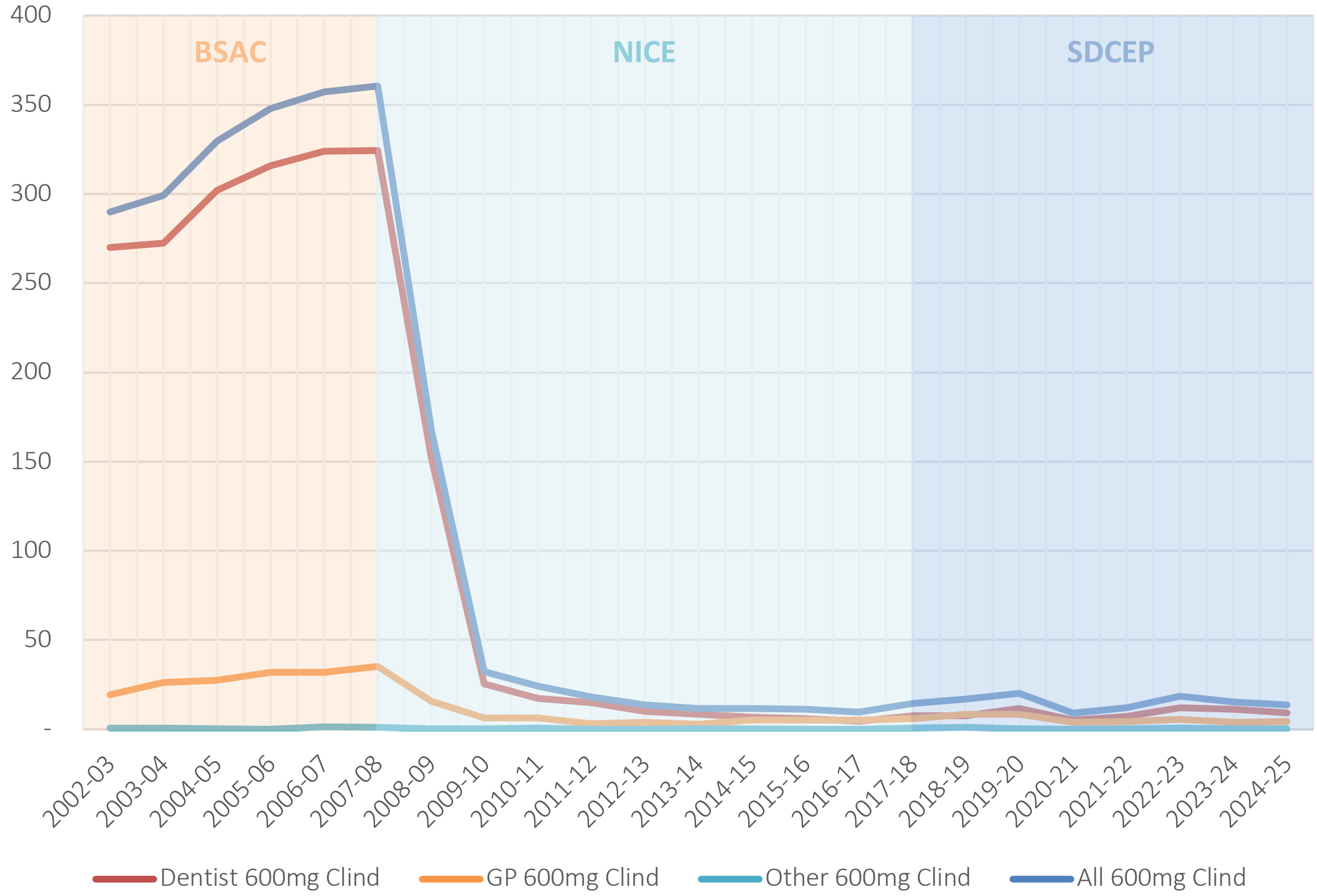

BSAC = British Society for Antimicrobial Chemotherapy guidance, NICE = National Institute for Health and Care Excellence guidance, SDCEP = Scottish Dental Clinical Effectiveness Programme implementation advice for the NICE guidelines, AP = antibiotic prophylaxis, Clind = clindamycin AP, GP = general medical practitioner, Other = pharmacist, nurse practitioner or other prescriber

Figure S2(c). Scottish AP prescribing/million of population by type of antibiotic

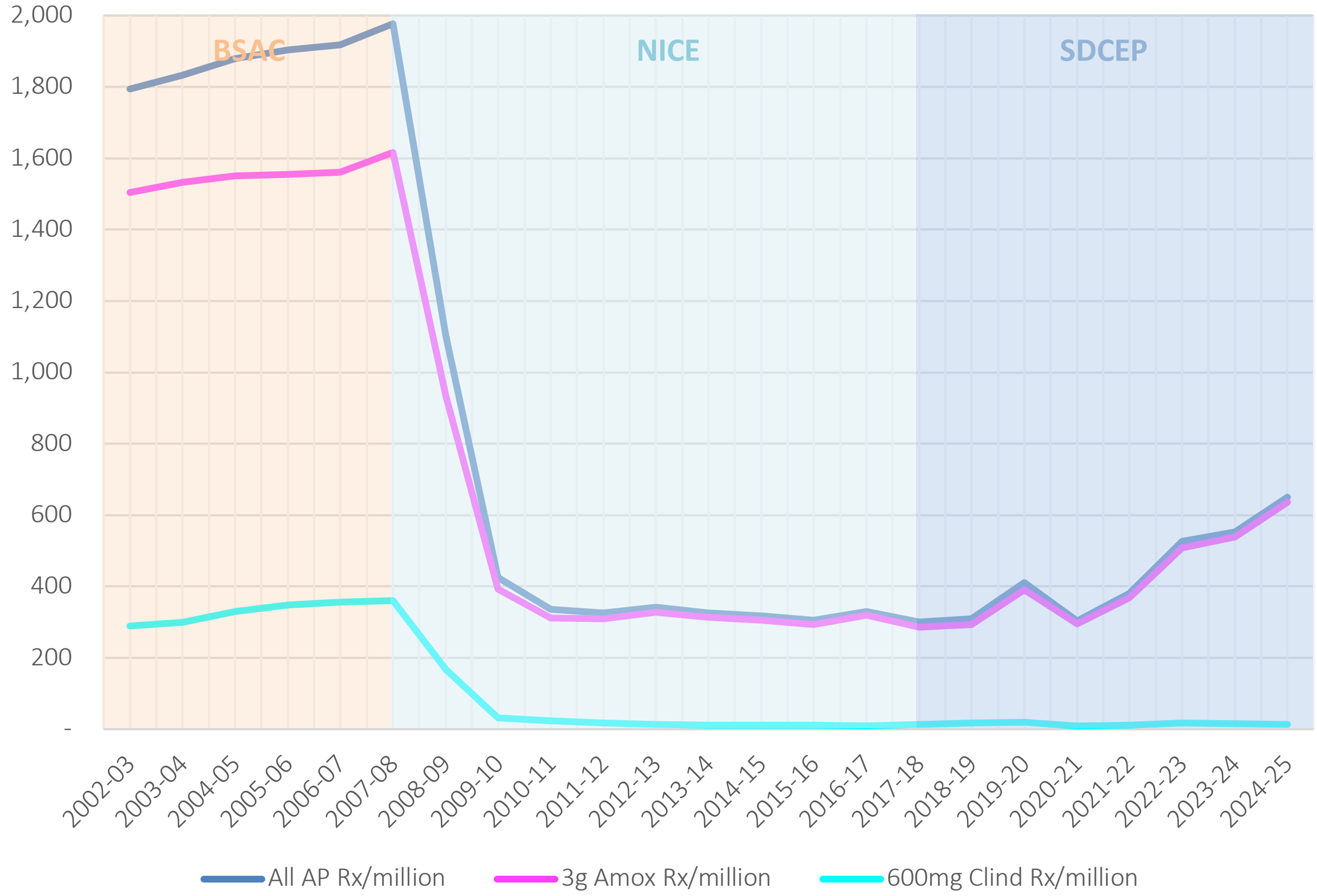

BSAC = British Society for Antimicrobial Chemotherapy guidance, NICE = National Institute for Health and Care Excellence guidance, SDCEP = Scottish Dental Clinical Effectiveness Programme implementation advice for the NICE guidelines, AP = antibiotic prophylaxis, Amox = amoxicillin AP, Clind = Clindamycin AP

Figure S2(d). Scottish AP prescribing/million of population by prescriber type

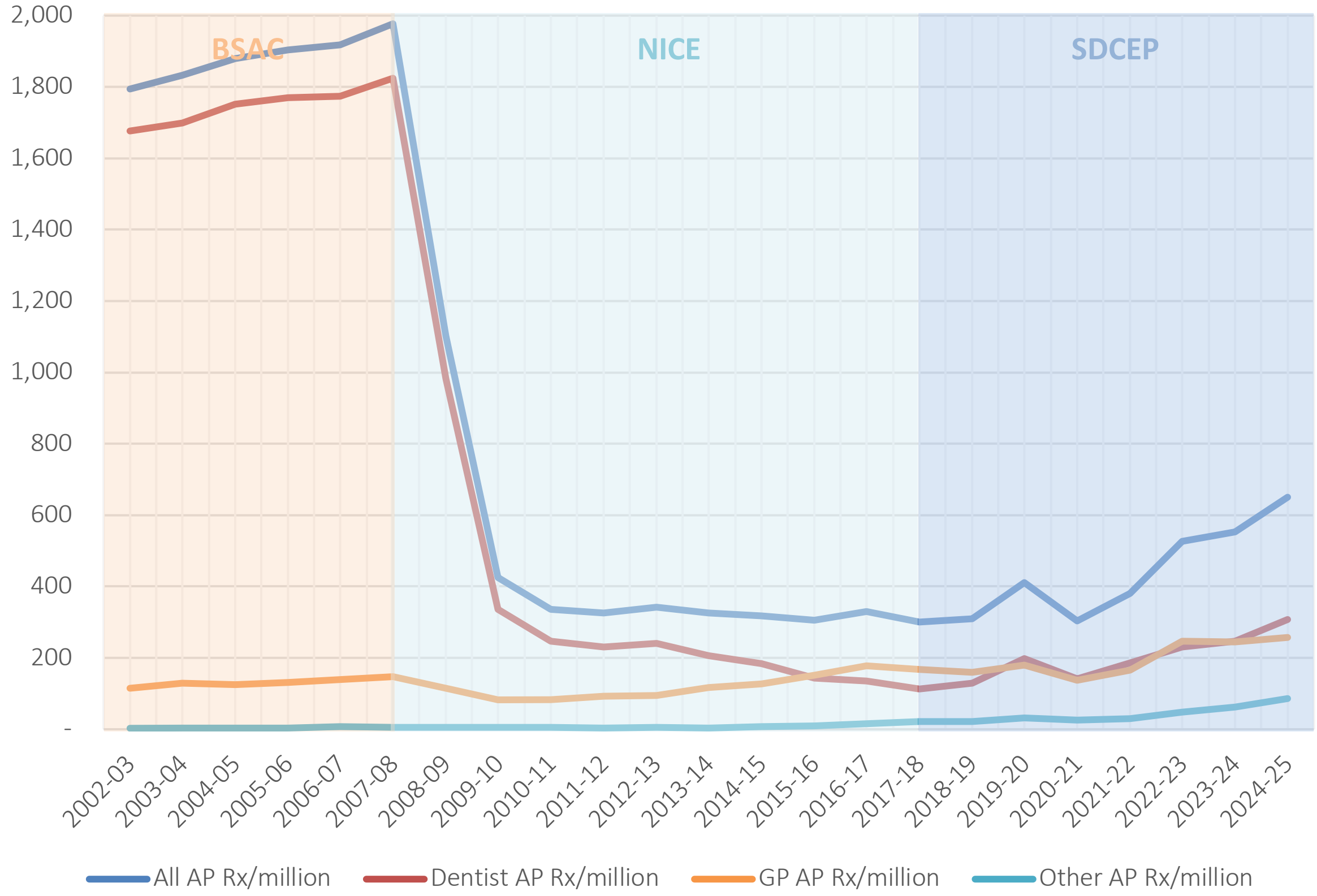

BSAC = British Society for Antimicrobial Chemotherapy guidance, NICE = National Institute for Health and Care Excellence guidance, SDCEP = Scottish Dental Clinical Effectiveness Programme implementation advice for the NICE guidelines, AP = antibiotic prophylaxis, GP = general medical practitioner, Other = pharmacist, nurse practitioner or other prescriber

Figure S3 A comparison of AP prescribing by dentists and GP's in England and Scotland

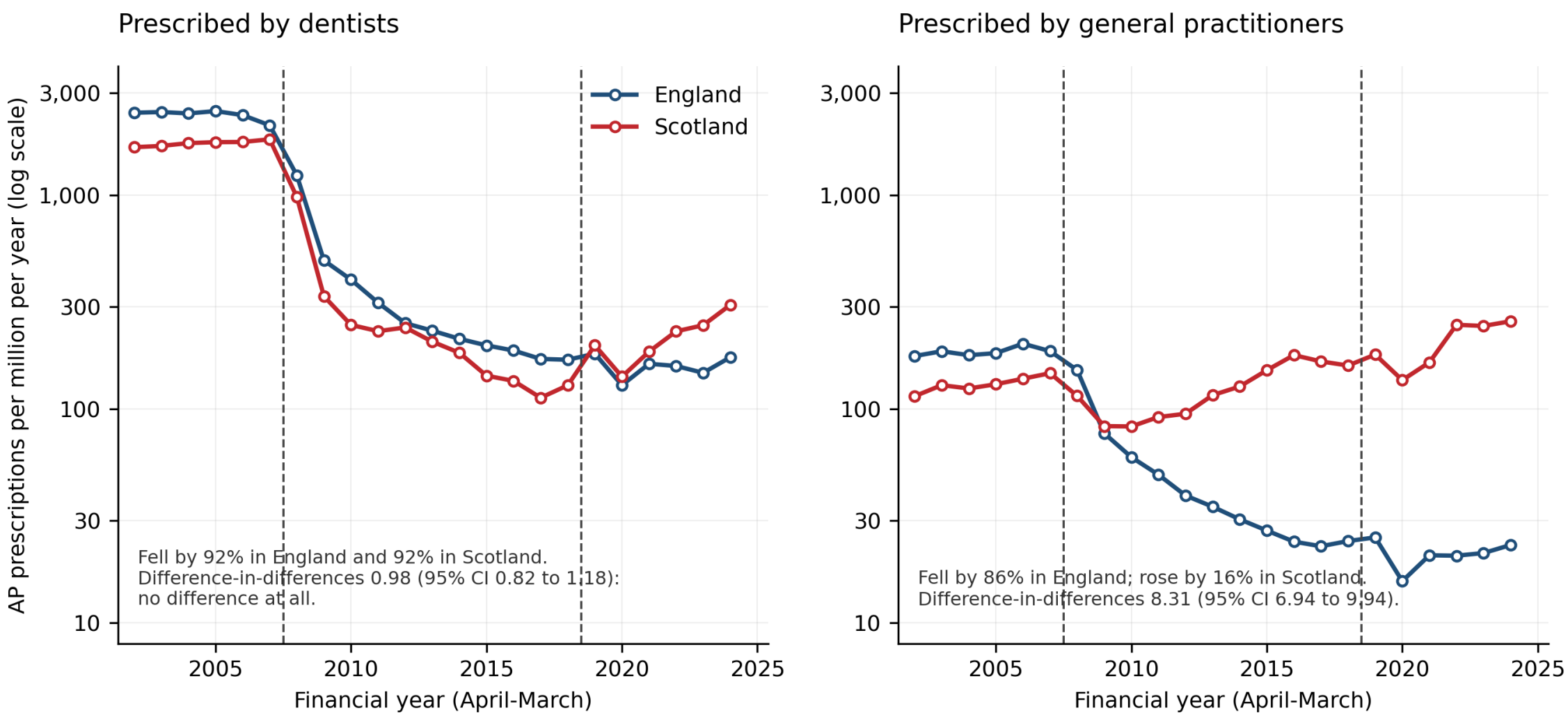

Dashed vertical lines mark publication of NICE CG64 (April 2008) and the SDCEP implementation advice (Aug 2018)

Figure S4. Observed IE admissions in England vs continuation of the pre-NICE trend.

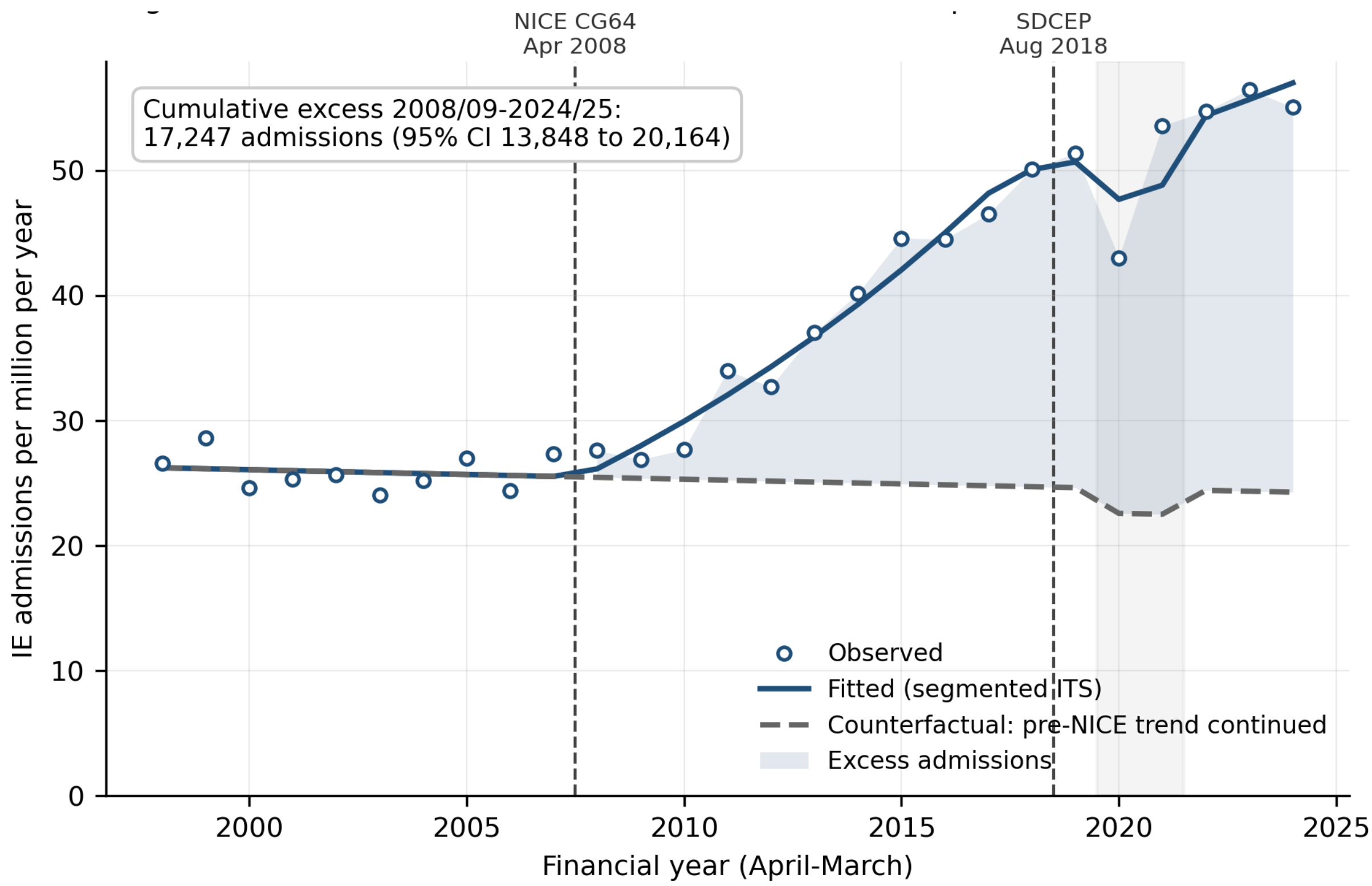

Detail of the post-implementation period from 2010/11 on a linear scale with independent y-axes. Fitted lines and confidence bands are from the model fitted to the full series.

Table S5. Controlled interrupted time series (segmented difference-in-differences) analysis.

Country-specific quantities are shown first, followed by the Scotland-versus-England interaction terms, which are the difference-in-differences estimates.

Estimate	IRR	95% CI	p
**AP prescriptions**			
England pre-NICE annual trend	0·959	0·899 to 1·023	0·207
England post-NICE annual trend	0·891	0·854 to 0·930	<0·0001
England post-SDCEP annual trend	0·990	0·921 to 1·064	0·782
Scotland pre-NICE annual trend	0·993	0·931 to 1·060	0·837
Scotland post-NICE annual trend	0·987	0·945 to 1·031	0·554
Scotland post-SDCEP annual trend	1·096	1·019 to 1·179	0·014
England NICE level change	0·201	0·133 to 0·304	<0·0001
England NICE slope change	0·929	0·860 to 1·004	0·064
England level change at 2019/20	1·352	0·942 to 1·942	0·102
England slope change from 2019/20	1·111	1·021 to 1·208	0·014
Scotland NICE level change	0·186	0·123 to 0·282	<0·0001
Scotland NICE slope change	0·994	0·919 to 1·075	0·876
Scotland SDCEP level change	1·337	0·927 to 1·927	0·120
Scotland SDCEP slope change	1·110	1·020 to 1·209	0·016
DiD baseline (Scotland vs England, series start)	0·673	0·508 to 0·890	0·005
DiD pre-NICE trend (parallel-trends test)	1·036	0·945 to 1·135	0·456
DiD NICE level	0·926	0·514 to 1·667	0·797
DiD NICE slope	1·070	0·958 to 1·194	0·231
DiD SDCEP level	0·988	0·591 to 1·653	0·964
DiD SDCEP slope	1·000	0·887 to 1·127	0·995
DiD post-NICE trend (Scotland vs England)	1·108	1·043 to 1·177	0·001
DiD post-SDCEP trend (Scotland vs England)	1·107	0·999 to 1·227	0·053

Estimate	IRR	95% CI	p
**IE admissions**			
England pre-NICE annual trend	0·997	0·986 to 1·008	0·598
England post-NICE annual trend	1·070	1·059 to 1·082	<0·0001
England post-SDCEP annual trend	1·024	0·999 to 1·049	0·056
Scotland pre-NICE annual trend	1·005	0·984 to 1·027	0·651
Scotland post-NICE annual trend	1·045	1·024 to 1·067	<0·0001
Scotland post-SDCEP annual trend	1·034	0·989 to 1·081	0·140
England NICE level change	1·027	0·939 to 1·123	0·565
England NICE slope change	1·073	1·057 to 1·090	<0·0001
England level change at 2019/20	0·918	0·820 to 1·028	0·140
England slope change from 2019/20	0·957	0·932 to 0·982	0·001
Scotland NICE level change	0·877	0·736 to 1·045	0·143
Scotland NICE slope change	1·040	1·009 to 1·071	0·010
Scotland SDCEP level change	0·874	0·706 to 1·082	0·216
Scotland SDCEP slope change	0·990	0·942 to 1·039	0·675
DiD baseline (Scotland vs England, series start)	0·899	0·789 to 1·024	0·108
DiD pre-NICE trend (parallel-trends test)	1·008	0·984 to 1·033	0·520
DiD NICE level	0·854	0·702 to 1·040	0·117
DiD NICE slope	0·969	0·937 to 1·002	0·062
DiD SDCEP level	0·952	0·747 to 1·212	0·687
DiD SDCEP slope	1·035	0·979 to 1·094	0·232
DiD post-NICE trend (Scotland vs England)	0·976	0·954 to 0·999	0·043
DiD post-SDCEP trend (Scotland vs England)	1·010	0·960 to 1·063	0·696

Table S6. Tests of the parallel-trends assumption.

Values are the ratio of the Scottish to the English annual trend over the window shown; a value of 1 indicates parallel trends. The prescribing series were not on parallel trends before either interruption; the IE series were parallel before NICE, and immediately before SDCEP Scottish admissions were if anything rising slightly faster than English ones.

Comparison	Ratio	95% CI	p
IE, pre-NICE (1998/99-2007/08)	1·008	0·984 to 1·032	0·513
IE, post-NICE / pre-SDCEP (2009/10-2017/18)	0·990	0·965 to 1·016	0·451
IE, immediately pre-SDCEP (2011/12-2018/19)	1·020	0·992 to 1·048	0·170
AP, pre-NICE (2002/03-2007/08)	1·037	1·019 to 1·056	<0·0001
AP, post-NICE / pre-SDCEP (2010/11-2017/18)	1·108	1·081 to 1·136	<0·0001
AP, immediately pre-SDCEP (2014/15-2017/18)	1·067	1·043 to 1·091	<0·0001

Table S7. IE and AP annual trends in England and Wales immediately preceding the SDCEP advice

Annual trend in each country over the windows immediately preceding the SDCEP advice, shown so that each country's own pre-intervention trajectory can be judged independently of the difference between them.

Estimate	IRR	95% CI	p
IE, immediately pre-SDCEP: England annual trend	1·063	1·052 to 1·073	<0·0001
IE, immediately pre-SDCEP: Scotland annual trend	1·083	1·056 to 1·112	<0·0001
IE, immediately pre-SDCEP: Ratio of trends (Scotland vs England)	1·020	0·992 to 1·048	0·170
AP, immediately pre-SDCEP: England annual trend	0·929	0·922 to 0·937	<0·0001
AP, immediately pre-SDCEP: Scotland annual trend	0·991	0·970 to 1·013	0·417
AP, immediately pre-SDCEP: Ratio of trends (Scotland vs England)	1·067	1·043 to 1·091	<0·0001
AP, pre-SDCEP (longer window): England annual trend	0·922	0·918 to 0·926	<0·0001
AP, pre-SDCEP (longer window): Scotland annual trend	0·982	0·970 to 0·993	0·001
AP, pre-SDCEP (longer window): Ratio of trends (Scotland vs England)	1·064	1·051 to 1·077	<0·0001

Table S8. Sensitivity of the NICE-era two-period difference-in-differences to the choice of pre-intervention window

Post-intervention window fixed at 2014/15–2017/18. Negative binomial model with a log population offset, fitted to the individual country-year counts; ratio of rate ratios (Scotland versus England) with Newey–West 95% confidence intervals, as in Table 3.

AP prescribing

Pre-NICE window	Years	Ratio of rate ratios (95% CI)	England post/pre	Scotland post/pre
2002/03–2007/08	6	1·941 (1·738 to 2·168)	0·086	0·166
2003/04–2007/08	5	1·916 (1·711 to 2·145)	0·086	0·165
2004/05–2007/08 (as published)	4	1·883 (1·675 to 2·116)	0·087	0·163
2005/06–2007/08	3	1·855 (1·638 to 2·102)	0·087	0·162
2006/07–2007/08	2	1·789 (1·589 to 2·013)	0·090	0·161
2002/03–2005/06 (earliest four)	4	2·021 (1·843 to 2·216)	0·084	0·169
2002/03–2004/05 (earliest three)	3	2·031 (1·848 to 2·232)	0·084	0·171
2002/03–2003/04 (earliest two)	2	2·064 (1·887 to 2·257)	0·084	0·173

IE admissions

Pre-NICE window	Years	Ratio of rate ratios (95% CI)	England post/pre	Scotland post/pre
1998/99–2007/08	10	0·751 (0·640 to 0·881)	1·698	1·275
2002/03–2007/08	6	0·739 (0·628 to 0·871)	1·716	1·269
2003/04–2007/08	5	0·731 (0·620 to 0·861)	1·717	1·255
2004/05–2007/08 (as published)	4	0·733 (0·625 to 0·860)	1·691	1·240
2005/06–2007/08	3	0·736 (0·626 to 0·866)	1·675	1·233
2006/07–2007/08	2	0·706 (0·591 to 0·843)	1·698	1·199
2002/03–2005/06 (earliest four)	4	0·758 (0·644 to 0·892)	1·725	1·307
2002/03–2004/05 (earliest three)	3	0·742 (0·631 to 0·873)	1·760	1·306

Pre-NICE window	Years	Ratio of rate ratios (95% CI)	England post/pre	Scotland post/pre
2002/03–2003/04 (earliest two)	2	0·753 (0·645 to 0·878)	1·768	1·331

Matched-length windows (pre- and post-intervention windows of equal length, each ending immediately before the next guideline event)

Window length	Windows compared	AP prescribing	IE admissions
2 years	2006/07–2007/08 vs 2016/17–2017/18	1·937 (1·768 to 2·121)	0·761 (0·683 to 0·848)
3 years	2005/06–2007/08 vs 2015/16–2017/18	1·918 (1·716 to 2·145)	0·743 (0·641 to 0·862)
4 years	2004/05–2007/08 vs 2014/15–2017/18	1·883 (1·675 to 2·116)	0·733 (0·625 to 0·860)
5 years	2003/04–2007/08 vs 2013/14–2017/18	1·849 (1·615 to 2·116)	0·738 (0·625 to 0·871)
6 years	2002/03–2007/08 vs 2012/13–2017/18	1·814 (1·556 to 2·116)	0·748 (0·620 to 0·902)

Table S9 — residual autocorrelation diagnostics

Model	n	NB dispersion	Durbin–Watson	Lag-1 residual autocorrelation	Ljung–Box p (lag 1)	Ljung–Box p (lag 2)
AP prescribing, England (segmented ITS)	23	1·54	3·01	−0·53	0·008	0·019
AP prescribing, Scotland (segmented ITS)	23	1·49	2·79	−0·40	0·040	0·121
AP prescribing, controlled ITS (both)	46	1·52	2·93	−0·47	0·001	0·004
IE admissions, England (segmented ITS)	27	1·43	2·54	−0·28	0·122	0·240
IE admissions, Scotland (segmented ITS)	27	1·39	2·24	−0·14	0·454	0·583
IE admissions, controlled ITS (both)	54	1·32	2·46	−0·23	0·079	0·213

STROBE Statement — Checklist of items to be included in reports of observational studies

Manuscript: "Guideline changes and their impact on antibiotic prophylaxis prescribing and endocarditis admissions in England and Scotland" (Thornhill, Prendergast, Dayer, Lockhart, Baddour)

Note on study type: the manuscript describes a quasi-experimental (natural experiment), controlled interrupted time series / difference-in-differences design comparing two national populations (England and Scotland) over time. This is an aggregate, population-level (ecological) design rather than a study of individually recruited participants, so several STROBE items written for cohort/case-control/cross-sectional studies of individuals (e.g. items 6, 13, 14) have been assessed against the closest available equivalent in the manuscript, with this noted explicitly in the "Assessment / Notes" column. 'Location in Manuscript' refers to sections/subsections, since fixed page numbers are not available at this review stage.

Item No.	STROBE Recommendation	Location in Manuscript	Assessment / Notes
**Title and abstract**			
1(a)	Indicate the study's design with a commonly used term in the title or the abstract	Title; Methods (Study design)	The title does not name the design, but the Abstract clearly states that the study uses an interrupted time series design with difference-in-differences estimation.
1(b)	Provide in the abstract an informative and balanced summary of what was done and what was found	Abstract (Background/Methods/Findings/Interpretation)	Structured abstract gives rationale, data sources, methods, key quantitative findings (with p-values) for both AP prescribing and IE admissions, and an appropriately cautious interpretation ("do not establish a causal relationship").
**Introduction**			
2	Explain the scientific background and rationale for the investigation being reported	Introduction (paras 1–5)	Well developed: history of BSAC/NICE/AHA/ESC guidance, the 2008 NICE decision and its rationale, and the divergent Scottish (SDCEP) pathway are all explained with citations.
3	State specific objectives, including any prespecified hypotheses	End of Introduction	"In this study we therefore aimed to compare the impact of the differing AP guidance in Scotland and England on AP prescribing and IE admissions." No formal a priori hypothesis is stated, though the direction of the expected association is implicit from the rationale.

Item No.	STROBE Recommendation	Location in Manuscript	Assessment / Notes
**Methods**			
4	Present key elements of study design early in the paper	Methods – "Study design" subsection	Explicitly labelled: quasi-experimental natural experiment, controlled interrupted time series with difference-in-differences, unit of analysis = financial year, two pre-specified outcomes.
5	Describe the setting, locations and relevant dates, including periods of recruitment, exposure, follow-up and data collection	Methods – "Antibiotic Prophylaxis Prescribing" and "Infective Endocarditis Admissions" subsections	Settings (England, Scotland), data sources (NHSBSA, Public Health Scotland, NHS England, FOI requests) and exact date ranges given (prescribing: Jan 2004–Mar 2025; IE admissions: Apr 1998–Mar 2025). Guideline dates (2008 NICE, 2018 SDCEP, 2024 NICE amendment) are given throughout.
6	Give the eligibility criteria, and the sources and methods of selection of participants	Methods – AP Prescribing / IE Admissions subsections	This is an ecological (population-level, not individual-participant) study, so classic "participant eligibility/selection" does not fully apply. The analogous units are defined precisely: AP = single 3g oral amoxicillin or 600mg oral clindamycin community prescriptions; IE admissions = hospital admissions with primary ICD-10 code I33.0.
7	Give diagnostic criteria, sources of data, and details of methods of assessment for each variable of interest	Methods - ICD-10 discussion	AP prescriptions and IE diagnosis (ICD-10 I33.0) are defined and diagnostic accuracy of I33.0 (sensitivity/specificity/PPV/NPV) is quantified.
8	For each variable of interest, give sources of data and details of methods of assessment (measurement). Describe comparability of assessment methods if more than one group	Methods (both subsections)	Data obtained via Freedom of Information Act from NHSBSA/Public Health Scotland/NHS England; identical prescription/diagnosis definitions applied to both nations, supporting comparability.

Item No.	STROBE Recommendation	Location in Manuscript	Assessment / Notes
9	Describe any efforts to address potential sources of bias	Methods model choice (NB2 vs Poisson) and in Limitations section	Coding reliability, independence of coding from the study/guideline changes, and a range of possible confounders (demographics, comorbidities, procedure rates) are discussed. Six sensitivity analyses (Methods) also address analytic/model-choice bias.
10	Explain how the study size was arrived at	Methods	As a whole-population, whole-period ecological study using complete national datasets, no sample-size/power calculation is presented or would be expected; and it states that – “Both national datasets were complete”
11	Explain how quantitative variables were handled in the analyses; describe which groupings were chosen and why	Methods (Model structure); Table 3 footnote	Annual counts modelled as rates per million (log population offset); four-year comparison “windows” used for difference-in-differences (rationale for the specific years is implicit in guideline timing).
12(a)	Describe all statistical methods, including those used to control for confounding	Methods – “Model structure”, “Autocorrelation”, “Counterfactual estimation” (+ Supplementary Methods)	Negative binomial (NB2) regression with log-link and log population offset; IRRs with 95% CI; controlled ITS and DiD estimation; COVID-19 adjustment; autocorrelation methods compared. Further technical detail is provided in the Supplementary Methods.
12(b)	Describe any methods used to examine subgroups and interactions	Results (prescriber-type and AP-type subgroup analyses); Supplementary tables/figures	Subgroup analysis by prescriber type (dentist/GP/other) and AP type is reported in Results/Discussion with reference to supplementary material.
12(c)	Explain how missing data were addressed	Methods – “Study design” subsection	“Both national datasets were complete” is stated; no missing-data methods needed.
12(d)	Cohort study – If applicable, explain how loss to follow-up was addressed	Not applicable	Not applicable to this aggregate national time-series design; there is no individual-level follow-up or attrition.

Item No.	STROBE Recommendation	Location in Manuscript	Assessment / Notes
12(e)	Describe any sensitivity analyses	Methods – “Sensitivity analyses” subsection	Six pre-specified sensitivity analyses listed (excluding COVID years, model without implementation period, restricted IE series, Poisson vs negative binomial, etc.), with results said to be unchanged in every case, detail in Supplementary Methods.
**Results**			
13(a)	Report the numbers of individuals at each stage of the study	Not applicable / partially addressed	No participant flow diagram, as this is a whole-population aggregate study rather than individually recruited participants. The total counts of admissions/prescriptions are given in Table 1, Table 3 and the Results text instead.
13(b)	Give reasons for non-participation at each stage	Not applicable	Not applicable – population-level administrative data with no individual recruitment or non-participation.
13(c)	Consider use of a flow diagram	Not included	A flow diagram is not used and is not essential for this ecological design, though a simple diagram of the two guideline “interruptions” (already partly shown as Figure 1's timeline) helps readers.
14(a)	Give characteristics of study participants and information on exposures and potential confounders	Limitations section (population demographics, comorbidity prevalence)	Because the unit of analysis is the nation-year rather than the individual, classic “participant characteristics” (Table 1 style demographics) are not presented for a cohort; instead, population-level comparators (median age, foreign-born proportion, valve intervention rates, CIED rates, haemodialysis, IV drug use, diabetes, imaging access) are described narratively in the Limitations, addressing potential confounding qualitatively rather than quantitatively/statistically.
14(b)	Indicate the number of participants with missing data for each variable of interest	Not applicable	Datasets described as complete; no missing-data-by-variable table required.

Item No.	STROBE Recommendation	Location in Manuscript	Assessment / Notes
14(c)	Cohort study – Summarise follow-up time	Methods (study period stated)	Study period (up to 27 years, financial-year units) is given, though this maps only loosely onto “follow-up time” given the ecological design.
15	Report numbers of outcome events or summary measures over time	Table 1; Figures 2 and 4; Results text	Annual AP prescription rates and IE-admission rates per million are reported in full for both nations across the study period.
16(a)	Give unadjusted estimates and, if applicable, confounder-adjusted estimates and their precision (e.g., 95% CI)	Tables 2 and 3; Results (Changes in AP prescribing / IE admissions)	Extensive IRRs, rate ratios and difference-in-differences estimates with 95% CIs and p-values are reported for every comparison of interest.
16(b)	Report category boundaries when continuous variables were categorised	Table 3 (four-year comparison windows); Methods (financial-year definition)	The pre/post comparison windows (e.g. 2004/05–07/08 vs 2014/15–17/18) and the financial-year convention are clearly defined.
16(c)	If relevant, translate relative risk estimates into absolute risk for a meaningful time period	Discussion (counterfactual estimation: excess admissions); Results	Absolute rates per million are reported throughout, and a counterfactual analysis converts the English trend into an estimated 17,247 excess IE admissions (95% CI 13,848–20,164) and translates rate differences into hypothetical excess IE deaths.
17	Report other analyses done – e.g. analyses of subgroups and interactions, and sensitivity analyses	Results (prescriber-type subgroup findings); Discussion; Supplementary Methods reference	Subgroup findings by prescriber type (dentist vs GP) are reported in the main text; sensitivity analysis outcomes are summarised (“conclusions were unchanged in every case”) with full detail deferred to Supplementary Methods.
**Discussion**			
18	Summarise key results with reference to study objectives	Discussion – “Summary of the findings”	Clearly restates the main prescribing and admissions findings for both nations in relation to the stated objective.
19	Discuss limitations of the study, taking into account sources of	Discussion – “Limitations” subsection	Detailed and candid: coding accuracy/generalisability, diagnostic sensitivity/specificity of ICD-10 I33.0,

Item No.	STROBE Recommendation	Location in Manuscript	Assessment / Notes
	potential bias or imprecision; discuss direction and magnitude of any potential bias		unmeasured confounders (with direction/magnitude discussed qualitatively for each), and lack of microbiological speciation data.
20	Give a cautious overall interpretation considering objectives, limitations, multiplicity of analyses, results from similar studies, and other relevant evidence	Discussion – "Possible implications"	Explicitly cautious: "The strong temporal association does not prove a causal relationship"; interpretation is anchored to the parallel-trends check and cross-referenced to prior published evidence on AP effectiveness (refs 21–24).
21	Discuss the generalisability (external validity) of the study results	Limitations	These results "may not be generalizable to other populations" outside the UK situation.
**Other information**			
22	Give the source of funding and the role of the funders for the present study and, if applicable, for the original study on which the present article is based	Abstract ("Funding"); Acknowledgements	"This study was unfunded" is stated in both the abstract and Acknowledgements. Role-of-funder statement is not needed given no funding.
